# (*R*,*R*_Fc_,*S*_Ru_)-Chlorido(η^6^-*p*-cymene){1-[1-(diphenyl­phosphanyl)ethyl]-2-[2-(diphenyl­phosphanyl)phenyl]ferrocene-κ^2^ *P*,*P*′}ruthenium(II) hexa­fluorido­phosphate

**DOI:** 10.1107/S1600536811036506

**Published:** 2011-09-14

**Authors:** Raffael Schuecker, Walter Weissensteiner, Kurt Mereiter

**Affiliations:** a Institute of Organic Chemistry, University of Vienna, Währingerstrasse 38, A-1090 Vienna, Austria; b Institute of Chemical Technologies and Analytics, Vienna University of Technology, Getreidemarkt 9/164SC, A-1060 Vienna, Austria

## Abstract

The asymmetric unit of the title compound, [FeRuCl(C_5_H_5_)(C_10_H_14_)(C_37_H_31_P_2_)]PF_6_, contains two independent, geometrically similar Ru^II^ complexes of a chiral ferrocenyldiphosphane with piano-stool coordination through the η^6^-bound *p*-cymene ligand, two chelating phospho­rus donor atoms, and an *exo*-oriented chloride ion. The mean bond lengths of the two Ru complexes are Ru—C = 2.276 Å, Ru—P = 2.3816 Å, and Ru—Cl = 2.3924 Å. Both chloride ligands form only intra­molecular C—H⋯Cl inter­actions. Seven weak inter­molecular C—H⋯F inter­actions involving mainly arene H atoms consolidate the crystal packing, which reveals an approximate *c*/2 pseudo-translation relating the two independent Ru complex mol­ecules.

## Related literature

For general information on ferrocene-based diphosphanes and their applications in asymmetric catalysis, see: Togni (1996 ▶); Blaser *et al.* (2002 ▶, 2007 ▶); Dai & Hou (2010 ▶); Solvias (2011 ▶). For the synthesis, coordination behaviour and use of Walphos-type ligands in asymmetric catalysis, see: Weissensteiner *et al.* (2002 ▶); Sturm *et al.* (2003 ▶); Wang *et al.* (2008 ▶). For crystal structures with Walphos-type ligands, see: Moberg *et al.* (2007 ▶); Maddox *et al.* (2008 ▶). For crystal structures of Ru(II)–(*p*-cymene) complexes with non-ferrocenyl phosphane ligands, see: Jensen *et al.* (1998 ▶); Lalrempuia *et al.* (2003 ▶); Chaplin & Dyson (2007 ▶). For a description of the Cambridge Structural Database, see: Allen (2002 ▶).
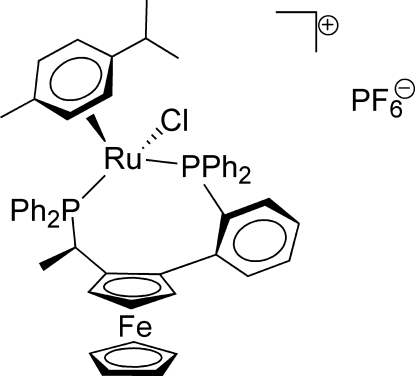


## Experimental

### 

#### Crystal data

[FeRuCl(C_5_H_5_)(C_10_H_14_)(C_37_H_31_P_2_)]PF_6_
*M* *_r_* = 1074.20
Monoclinic, 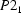

*a* = 12.7479 (8) Å
*b* = 15.8091 (10) Å
*c* = 22.8897 (15) Å
β = 97.378 (2)°
*V* = 4574.8 (5) Å^3^
*Z* = 4
Mo *K*α radiation
μ = 0.87 mm^−1^
*T* = 100 K
0.42 × 0.33 × 0.24 mm

#### Data collection

Bruker Kappa APEXII CCD diffractometer
Absorption correction: multi-scan (*SADABS*; Bruker, 2008 ▶) *T*_min_ = 0.74, *T*_max_ = 0.81
84544 measured reflections
26641 independent reflections
25880 reflections with *I* > 2σ(*I*)
*R*_int_ = 0.024

#### Refinement

*R*[*F* ^2^ > 2σ(*F* ^2^)] = 0.027
*wR*(*F* ^2^) = 0.069
*S* = 1.02
26641 reflections
1154 parameters
1 restraint
H-atom parameters constrained
Δρ_max_ = 1.32 e Å^−3^
Δρ_min_ = −0.29 e Å^−3^
Absolute structure: Flack (1983 ▶), 12869 Friedel pairs
Flack parameter: −0.005 (6)

### 

Data collection: *APEX2* (Bruker, 2008 ▶); cell refinement: *SAINT* (Bruker, 2008 ▶); data reduction: *SAINT* and *XPREP* (Bruker, 2008 ▶); program(s) used to solve structure: *SHELXS97* (Sheldrick, 2008 ▶); program(s) used to refine structure: *SHELXL97* (Sheldrick, 2008 ▶); molecular graphics: *Mercury* (Macrae *et al.*, 2006 ▶); software used to prepare material for publication: *PLATON* (Spek, 2009 ▶) and *publCIF* (Westrip, 2010 ▶).

## Supplementary Material

Crystal structure: contains datablock(s) I, global. DOI: 10.1107/S1600536811036506/kp2346sup1.cif

Structure factors: contains datablock(s) I. DOI: 10.1107/S1600536811036506/kp2346Isup2.hkl

Additional supplementary materials:  crystallographic information; 3D view; checkCIF report

## Figures and Tables

**Table 1:** Selected bond lengths (Å)

Ru1—P1	2.3896 (5)
Ru1—P2	2.3810 (5)
Ru1—Cl1	2.3967 (4)
Ru1—C43	2.301 (2)
Ru1—C44	2.262 (2)
Ru1—C45	2.253 (2)
Ru1—C46	2.329 (2)
Ru1—C47	2.250 (2)
Ru1—C48	2.257 (2)
Fe1—C1	2.093 (2)
Fe1—C2	2.099 (2)
Fe1—C3	2.050 (2)
Fe1—C4	2.039 (2)
Fe1—C5	2.038 (2)
Fe1—C6	2.077 (2)
Fe1—C7	2.084 (2)
Fe1—C8	2.058 (2)
Fe1—C9	2.030 (2)
Fe1—C10	2.048 (2)
Ru2—P3	2.3834 (5)
Ru2—P4	2.3725 (5)
Ru2—Cl2	2.3880 (5)
Ru2—C95	2.320 (2)
Ru2—C96	2.261 (2)
Ru2—C97	2.256 (2)
Ru2—C98	2.318 (2)
Ru2—C99	2.235 (2)
Ru2—C100	2.272 (2)
Fe2—C53	2.094 (2)
Fe2—C54	2.096 (2)
Fe2—C55	2.052 (2)
Fe2—C56	2.034 (2)
Fe2—C57	2.039 (2)
Fe2—C58	2.072 (2)
Fe2—C59	2.083 (2)
Fe2—C60	2.055 (2)
Fe2—C61	2.040 (2)
Fe2—C62	2.048 (2)

**Table 2:** Hydrogen-bond geometry (Å, °)

*D*—H⋯*A*	*D*—H	H⋯*A*	*D*⋯*A*	*D*—H⋯*A*
C20—H20⋯Cl1	0.95	2.69	3.499 (2)	143
C72—H72⋯Cl2	0.95	2.56	3.389 (2)	145
C13—H13⋯F3^i^	0.95	2.51	3.076 (2)	118
C22—H22⋯F5^ii^	0.95	2.40	3.312 (3)	161
C34—H34⋯F6^iii^	0.95	2.48	3.161 (3)	129
C51—H51*C*⋯F9^iv^	0.98	2.54	3.422 (4)	149
C60—H60⋯F6	0.95	2.55	3.380 (3)	146
C65—H65⋯F11^v^	0.95	2.53	3.395 (3)	152
C74—H74⋯F7^vi^	0.95	2.50	3.130 (3)	124

Symmetry codes: (i) 
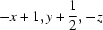
; (ii) 
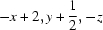
; (iii) 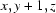; (iv) 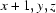; (v) 
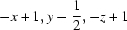
; (vi) 
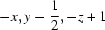
.

**Fig. 1.**
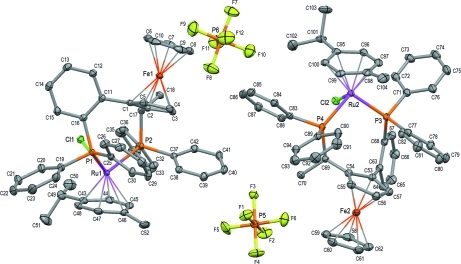
The asymmetric unit of (I) with displacement ellipsoids at the 50% probability level. Hydrogen atoms omitted for clarity.

**Fig. 2.**
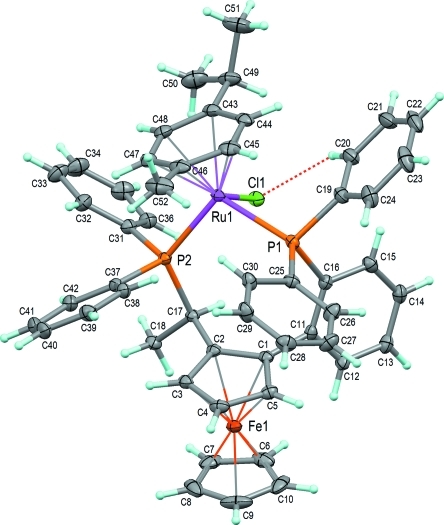
View of the complex of Ru1 with displacement ellipsoids for the non-hydrogen atoms at the 50% probability level and hydrogen atoms as spheres of arbitrary radius. The intramolecular C—H···Cl interaction (dashed line) has C20···Cl1 = 3.449 (2) Å.

**Fig. 3.**
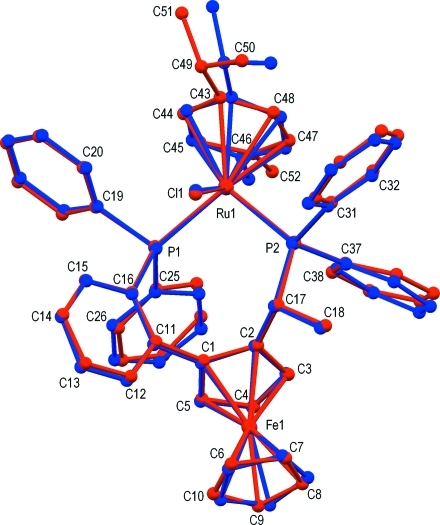
Superposition plot of the complexes of Ru1 (red) and Ru2 (blue). The r.m.s. deviation of the fitted atom pairs Ru1/Ru2, P1/P3, P2/P4, Cl1/Cl2, and Fe1/Fe2 is 0.054 Å. The largest positional difference is 0.803 Å for C52 and its equivalent C104 (in blue). Only selected atoms of the Ru1 complex are labelled. H-atoms omitted for clarity.

**Fig. 4.**
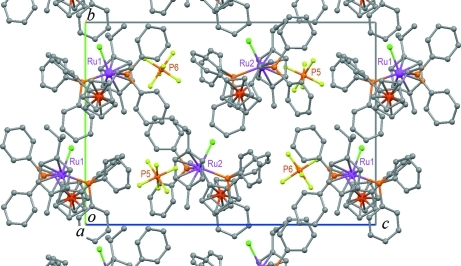
Packing diagram of (I) viewed along the a-axis. H-atoms omitted for clarity.

**Fig. 5.**
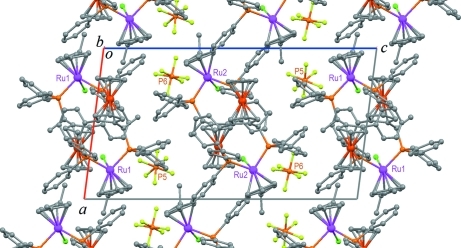
Packing diagram of (I) viewed along the b-axis. H-atoms omitted for clarity.

## supplementary crystallographic information

### Comment

Ferrocenyl-based diphosphines with an asymmetric carbon atom attached to 1,2-disubstituted planar chiral ferrocenes have emerged as valuable ligands of platinum group metals applied in a variety of important catalytical asymmetric transformations (Togni, 1996; Blaser *et al.*, 2002; Blaser *et al.*, 2007; Dai & Hou, 2010). One of their advantages is that they can be modularly designed and synthesised to give larger ligand families like the well known Josiphos ligands, one of which serves the highly efficient industrial production of the enantiopure herbicide (*S*)-metolachlor in thousands of tons per year (Blaser *et al.*, 2007). The diphosphine ligand of the title compound belongs to another such family known as the Walphos ligands (Weissensteiner *et al.*, 2002; Sturm *et al.*, 2003; Wang *et al.*, 2008). Within this family, the ligand of the title compound has obtained the designation Walphos SL—W002–1 [chemical name: (*R*,*R*_Fc_)-[1-[1-(diphenylphosphanyl)ethyl]-2-[2-(diphenylphosphanyl)phenyl]ferrocene]; Solvias, 2011], of which a CF_3_-substituted derivative (Walphos SL—W001–1) is now in use in a Rh catalysed key step of the industrial synthesis of the chiral hypertension drug aliskiren (Blaser *et al.*, 2007; Wang *et al.*, 2008). While in previous crystallographic research only mononuclear PdCl_2_ and PtCl_2_ complexes of Walphos-type ligands were structurally characterised (Wang *et al.*, 2008; Maddox *et al.*, 2008), it was now of interest to determine the crystal structure of a Ru half-sandwich complex of this ligand family. Since all attempts to crystallise a Ru-η^6^-arene complex of Walphos SL—W001–1 failed, the title compound, (I), a Ru(II) complex with *p*-cymene and the Walphos ligand SL—W002–1 (Scheme 1) was successfully crystallised and studied by X-ray diffraction.

The title compound, (I) crystallizes with two crystallographically independent Ru complexes of very similar overall geometry (Table 1 and Figs. 1, 2 and 3). In both molecules, the Ru(II) reveals a three-legged piano-stool coordination with the η^6^-bound *p*-cymene ligand, the two chelating phosphorus donor atoms, and an *exo*-oriented chloride ligand. The bond lengths of the two Ru complexes are in the ranges Ru—C = 2.235 (2) – 2.329 (2) Å (mean value 2.276 Å), Ru—P = 2.3725 (5) – 2.3896 (5) Å (mean value 2.3816 Å), and Ru—Cl = 2.3880 (5) – 2.3967 (4) Å (mean value 2.3924 Å). The bond angles P—Ru—P (102.34 (2)° and 101.02 (2)°) are systematically larger than the angles P—Ru—Cl (80.41 (2) – 83.26 (2)°). These values correspond well with those found in [Ru(*p*-cymene)(PPh_3_)_2_Cl](BF_4_) [refcode ESCOLUT (Lalrempuia *et al.*, 2003)] and related complexes [refcodes HAJCUQ (Jensen *et al.*, 1998), SIGJUO and SIGKEZ (Chaplin & Dyson, 2007); Cambridge Structural Database, Version 5.31, with Aug. 2010 updates; Allen, 2002]. Interestingly, they all, including (I), have the isopropyl group of the *p*-cymene ligand placed approximately over the Ru—Cl bond. It appears that this feature is not due to electronic rather than steric reasons, namely least steric congestion by the P-bonded phenyl rings in the vicinity of Cl. The P-metal-P triangle in (I) adopts an *exo*-orientation, which turns its tip (Ru) away from the line through the centroids of the two ferrocene cyclopentadienyl rings. In the PdCl_2_ and PtCl_2_ complexes of Walphos-type ligands [refcodes JOFYAF and JOFJEY (Maddox *et al.*, 2008); QODHIB, QODHOH, QODHUN, QOFRIN (Wang *et al.*, 2008)] this triangle as part of a distorted square-planar metal-P_2_Cl_2_ coordination is with its tip (Pd, Pt) turned toward this line. This change in the metal position is enabled by a significant flexibility of Walphos ligands, which normally form eight-membered chelate rings. Evidence for this flexibility is provided by tetranuclear Ru cluster complexes, where Walphos ligands bridge the two Ru atoms with a P—P bite distance of *ca* 5.6 Å (Moberg *et al.*, 2007), whereas in mononuclear complexes like (I) this distance is about 3.7 Å.

The crystal structure bears some pseudosymmetry because the complexes of Ru1 located near *z*≈ 0, 1, *etc* (Figs. 4 and 5) show similar orientations and spatial arrangements like the complexes of Ru2 located near *z*≈ 1/2, 3/2, *etc*, leading to a pseudo-translation of *c*/2. However, in closer detail the differences between the structure segements near *z*≈ 0 and *z*≈ 1/2 are considerable, as can be seen for instance by comparing the *y*-coordinates of Ru1 and Ru2(-*x*, *y* + 1/2, -*z*), 0.749937 (8) *versus* 0.781768 (9), corresponding to a difference of 0.50 Å, or the phenyl ring positions of Ru1 and Ru2 complexes relative to the unit cell box, *e.g.* along the blue *z*-axis (Fig. 5). Moreover, the spatial orientations of the PF_6_ octahedra of P5 and P6 are entirely different and violating the *c*/2 pseudo-translation. Apart from this pseudosymmetry, the crystal packing of (I) bears no especially remarkable features. Both chloride ligands form only intramolecular C—H···Cl interactions and seven weak intermolecular C—H···F interactions involving mainly arene hydrogen atoms consolidate the crystal packing (Table 2).

### Experimental

A suspension of [RuCl_2_(*p*-cymene)]_2_ (23.3 mg, 0.04 mmol) and silver nitrate (12.9 mg, 0.08 mmol) in methanol (2 mL) was stirred at ambient temperature for 1 h. After filtration the filtrate was added to a suspension of Walphos SL—W002–1 (50.0 mg, 0.08 mmol) in methanol (2 mL). The resulting mixture was stirred for two hours resulting in a red solution. This reaction mixture was again filtered and  a solution of ammonium hexafluorophosphate (37.1 mg, 0.2 mmol) in methanol (1 mL) was added to the filtrate. Partial removal of the solvent under reduced pressure induced precipitation of the orange complex, which was filtered off, washed with cold methanol and diethyl ether and dried *in vacuo* (42 mg; 59%). Crystals suitable for X-ray diffraction were grown by vapour diffusion of hexane into a saturated dichloromethane solution.

^1^H NMR (400 MHz, acetone-d_6_): *δ* 0.45 (d, *J* = 6.8 Hz, 3H, H50), 0.99 (d, *J* = 7.1 Hz, 3H, H51), 1.60 (dd, *J* = 7.18 Hz, *J* = 12.21 Hz, 3H, H12), 2.17 (br s, 3H,H52), 2.21–2.30 (m, 1H, H49), 2.36 (br s, 1H, H3), 2.94 (br s, 1H, H5), 3.10 (dq, *J*_1_ = *J*_2_ = 7.03 Hz, 1H, H11), 3.10 (dd, *J*_1_ = *J*_2_ = 2.50 Hz, 1H, H4), 4.09 (s, 1H, H6–H10), 4.39 (br s, 1H, H44), 4.63 (br s, 1H, H48), 5.07 (br s, 1H, H45), 5.89 (br s, 1H, H47), 7.26–2.36 (m, 2H, H26+H30),7.36–7.43 (m, 1H, H16), 7.97–8.06 (m, 1H, H17), 8.37–8.45 (m, 1H, H14); ^13^C{^1^H} NMR (100.6 MHz, CDCl_3_): *δ* 18.5 (br s, C52), 19.2 (br s, C50), 21.7 (br d, *J* = 4.5 Hz, C12), 22.3 (s, C51), 28.3 (d, *J* = 18.4 Hz, C11), 30.9 (s, C49), 65.2 (br d, *J* = 2.3 Hz, C3), 65.6 (br s, C4), 70.0 (br s, 5 C, C6–C10), 71.0 (s, C5), 90.2 (br s, C2), 90.6 (br d, *J* = 9.1 Hz, C44), 95.6 (C1), 96.4 (s, C47), 90.6 (d, *J* = 4.2 Hz, C45), 126.4 (d, *J* = 8.8 Hz, C16), 128.4 (d, *J* = 8.8 Hz,2 C, Ph-*meta*), 128.4 (d, *J* = 10.3 Hz, 2 C, Ph-*meta*), 129.9 (d, *J* = 39.8 Hz, Ph-*ipso*),130.3 (d, *J* = 2.6 Hz, C15), 130.8 (d, *J* = 2.6 Hz, Ph-*para*), 131.2 (d, *J* = 2.3 Hz, Ph-*para*), 132.2 (d, *J* = 2.3 Hz, Ph-*para*), 133.0 (d, *J* = 2.3 Hz, Ph-*para*), 135.6 (d, *J* = 5.1 Hz, C17), 136.6 (br d, *J* = 13.0 Hz, 2 C, Ph-*ortho*), 136.7 (d, *J* = 44.8 Hz, Ph-*ipso*), 138.5 (d, *J* = 11.1 Hz, C13), 139.5 (br d, *J* = 8.7 Hz, C14), 141.6 (d, *J* = 44.7 Hz, Ph-*ipso*); ^31^P NMR (162 MHz, acetone-d_6_): *δ* -143.0 (PF_6_), 26.6 (d, *J* = 47.7 Hz, P1), 42.1 (d, *J* = 49.0 Hz, P2).

### Refinement

H atoms were located in a difference Fourier map, placed in calculated positions and thereafter treated as riding, C—H = 0.95 – 1.00 Å. A torsional parameter was refined for each methyl group. *U*_iso_(H) = 1.2*U*_eq_(C) for CH groups; *U*_iso_(H) = 1.5*U*_eq_(C) for CH_3_ groups.

### Crystal data

**Table:**

[FeRuCl(C_5_H_5_)(C_10_H_14_)(C_37_H_31_P_2_)]PF_6_	*F*(000) = 2192
*M**_r_* = 1074.20	*D*_x_ = 1.560 Mg m^−^^3^
Monoclinic, *P*2_1_	Mo *K*α radiation, λ = 0.71073 Å
*a* = 12.7479 (8) Å	Cell parameters from 8771 reflections
*b* = 15.8091 (10) Å	θ = 2.2–30.0°
*c* = 22.8897 (15) Å	µ = 0.87 mm^−^^1^
β = 97.378 (2)°	*T* = 100 K
*V* = 4574.8 (5) Å^3^	Block, orange
*Z* = 4	0.42 × 0.33 × 0.24 mm

### Data collection

**Table:**

Bruker Kappa APEXII CCD diffractometer	26641 independent reflections
Radiation source: fine-focus sealed tube	25880 reflections with *I* > 2σ(*I*)
graphite	*R*_int_ = 0.024
φ and ω scans	θ_max_ = 30.0°, θ_min_ = 2.2°
Absorption correction: multi-scan (*SADABS*; Bruker, 2008)	*h* = −17→17
*T*_min_ = 0.74, *T*_max_ = 0.81	*k* = −22→22
84544 measured reflections	*l* = −32→32

### Refinement

**Table:**

Refinement on *F*^2^	Secondary atom site location: difference Fourier map
Least-squares matrix: full	Hydrogen site location: inferred from neighbouring sites
*R*[*F*^2^ > 2σ(*F*^2^)] = 0.027	H-atom parameters constrained
*wR*(*F*^2^) = 0.069	*w* = 1/[σ^2^(*F*_o_^2^) + (0.0467*P*)^2^ + 0.788*P*] where *P* = (*F*_o_^2^ + 2*F*_c_^2^)/3
*S* = 1.02	(Δ/σ)_max_ = 0.002
26641 reflections	Δρ_max_ = 1.32 e Å^−^^3^
1154 parameters	Δρ_min_ = −0.29 e Å^−^^3^
1 restraint	Absolute structure: Flack (1983), 12869 Friedel pairs
Primary atom site location: structure-invariant direct methods	Flack parameter: −0.005 (6)

### Special details

**Table:**

Geometry. All e.s.d.'s (except the e.s.d. in the dihedral angle between two l.s. planes) are estimated using the full covariance matrix. The cell e.s.d.'s are taken into account individually in the estimation of e.s.d.'s in distances, angles and torsion angles; correlations between e.s.d.'s in cell parameters are only used when they are defined by crystal symmetry. An approximate (isotropic) treatment of cell e.s.d.'s is used for estimating e.s.d.'s involving l.s. planes.
Refinement. Refinement of *F*^2^ against ALL reflections. The weighted *R*-factor *wR* and goodness of fit *S* are based on *F*^2^, conventional *R*-factors *R* are based on *F*, with *F* set to zero for negative *F*^2^. The threshold expression of *F*^2^ > σ(*F*^2^) is used only for calculating *R*-factors(gt) *etc*. and is not relevant to the choice of reflections for refinement. *R*-factors based on *F*^2^ are statistically about twice as large as those based on *F*, and *R*- factors based on ALL data will be even larger.

### Fractional atomic coordinates and isotropic or equivalent isotropic displacement parameters (Å^2^)

**Table:**

	*x*	*y*	*z*	*U*_iso_*/*U*_eq_	
Ru1	0.807431 (10)	0.749937 (8)	0.081291 (6)	0.01352 (3)	
Fe1	0.338025 (19)	0.633083 (16)	0.047262 (12)	0.01492 (5)	
Cl1	0.72029 (3)	0.87894 (3)	0.04842 (2)	0.01729 (8)	
P1	0.71915 (4)	0.70390 (3)	−0.01168 (2)	0.01436 (8)	
P2	0.67051 (3)	0.74241 (3)	0.142731 (19)	0.01308 (7)	
C1	0.47826 (13)	0.66341 (11)	0.01347 (8)	0.0148 (3)	
C2	0.49227 (13)	0.67440 (11)	0.07661 (8)	0.0144 (3)	
C3	0.46918 (14)	0.59441 (12)	0.10200 (8)	0.0169 (3)	
H3	0.4712	0.5830	0.1429	0.020*	
C4	0.44280 (15)	0.53501 (12)	0.05553 (9)	0.0202 (3)	
H4	0.4259	0.4771	0.0601	0.024*	
C5	0.44631 (14)	0.57768 (12)	0.00129 (9)	0.0179 (3)	
H5	0.4302	0.5534	−0.0368	0.022*	
C6	0.21079 (16)	0.71186 (15)	0.01984 (11)	0.0280 (4)	
H6	0.2138	0.7647	0.0004	0.034*	
C7	0.21814 (15)	0.70011 (16)	0.08070 (11)	0.0299 (5)	
H7	0.2274	0.7435	0.1096	0.036*	
C8	0.20961 (17)	0.61392 (18)	0.09174 (11)	0.0345 (6)	
H8	0.2114	0.5886	0.1295	0.041*	
C9	0.19786 (17)	0.57042 (15)	0.03724 (14)	0.0375 (6)	
H9	0.1910	0.5110	0.0319	0.045*	
C10	0.19818 (16)	0.63193 (19)	−0.00791 (10)	0.0333 (5)	
H10	0.1912	0.6214	−0.0491	0.040*	
C11	0.49411 (14)	0.72564 (11)	−0.03325 (7)	0.0147 (3)	
C12	0.40413 (14)	0.75633 (13)	−0.06836 (8)	0.0191 (3)	
H12	0.3365	0.7382	−0.0602	0.023*	
C13	0.40975 (16)	0.81195 (13)	−0.11438 (8)	0.0205 (3)	
H13	0.3469	0.8315	−0.1372	0.025*	
C14	0.50787 (16)	0.83900 (12)	−0.12696 (8)	0.0202 (3)	
H14	0.5130	0.8788	−0.1576	0.024*	
C15	0.59808 (15)	0.80728 (12)	−0.09433 (8)	0.0192 (3)	
H15	0.6651	0.8245	−0.1040	0.023*	
C16	0.59444 (13)	0.75057 (12)	−0.04738 (7)	0.0147 (3)	
C17	0.52844 (12)	0.75379 (12)	0.11013 (7)	0.0137 (3)	
H17	0.5239	0.8016	0.0813	0.016*	
C18	0.45701 (14)	0.77562 (13)	0.15755 (8)	0.0183 (3)	
H18A	0.3837	0.7814	0.1390	0.027*	
H18B	0.4805	0.8290	0.1767	0.027*	
H18C	0.4615	0.7304	0.1870	0.027*	
C19	0.80866 (15)	0.72828 (13)	−0.06670 (8)	0.0200 (3)	
C20	0.84836 (16)	0.81104 (14)	−0.06959 (9)	0.0247 (4)	
H20	0.8284	0.8533	−0.0436	0.030*	
C21	0.91681 (18)	0.83148 (17)	−0.11039 (11)	0.0316 (5)	
H21	0.9427	0.8877	−0.1122	0.038*	
C22	0.94754 (18)	0.77056 (18)	−0.14845 (10)	0.0355 (6)	
H22	0.9953	0.7846	−0.1756	0.043*	
C23	0.9081 (2)	0.68949 (18)	−0.14646 (10)	0.0354 (5)	
H23	0.9284	0.6477	−0.1726	0.043*	
C24	0.83808 (18)	0.66815 (14)	−0.10606 (9)	0.0267 (4)	
H24	0.8106	0.6123	−0.1056	0.032*	
C25	0.69419 (14)	0.59122 (11)	−0.02222 (8)	0.0166 (3)	
C26	0.63735 (16)	0.55861 (13)	−0.07382 (9)	0.0235 (4)	
H26	0.6124	0.5955	−0.1053	0.028*	
C27	0.61762 (16)	0.47251 (13)	−0.07884 (10)	0.0244 (4)	
H27	0.5798	0.4507	−0.1141	0.029*	
C28	0.65225 (17)	0.41791 (13)	−0.03314 (10)	0.0238 (4)	
H28	0.6368	0.3592	−0.0366	0.029*	
C29	0.70973 (18)	0.44934 (13)	0.01781 (9)	0.0239 (4)	
H29	0.7344	0.4123	0.0493	0.029*	
C30	0.73091 (16)	0.53539 (12)	0.02245 (8)	0.0195 (3)	
H30	0.7716	0.5565	0.0571	0.023*	
C31	0.69702 (14)	0.83032 (12)	0.19453 (8)	0.0176 (3)	
C32	0.76406 (16)	0.81879 (13)	0.24755 (9)	0.0217 (4)	
H32	0.7887	0.7636	0.2586	0.026*	
C33	0.79500 (18)	0.88706 (15)	0.28427 (10)	0.0292 (4)	
H33	0.8406	0.8781	0.3199	0.035*	
C34	0.75949 (18)	0.96803 (15)	0.26891 (11)	0.0307 (5)	
H34	0.7805	1.0146	0.2939	0.037*	
C35	0.69270 (18)	0.98045 (14)	0.21645 (11)	0.0274 (4)	
H35	0.6685	1.0358	0.2057	0.033*	
C36	0.66103 (15)	0.91247 (12)	0.17966 (9)	0.0198 (3)	
H36	0.6148	0.9217	0.1443	0.024*	
C37	0.66744 (13)	0.65209 (11)	0.19221 (8)	0.0150 (3)	
C38	0.69975 (15)	0.57303 (12)	0.17468 (8)	0.0175 (3)	
H38	0.7268	0.5676	0.1381	0.021*	
C39	0.69326 (16)	0.50170 (13)	0.20967 (9)	0.0227 (4)	
H39	0.7164	0.4484	0.1971	0.027*	
C40	0.65301 (17)	0.50860 (14)	0.26294 (9)	0.0253 (4)	
H40	0.6488	0.4602	0.2871	0.030*	
C41	0.61889 (16)	0.58683 (15)	0.28062 (9)	0.0242 (4)	
H41	0.5904	0.5917	0.3168	0.029*	
C42	0.62605 (15)	0.65815 (13)	0.24583 (8)	0.0191 (3)	
H42	0.6027	0.7114	0.2585	0.023*	
C43	0.95931 (14)	0.82975 (13)	0.09776 (9)	0.0210 (4)	
C44	0.96915 (14)	0.77033 (13)	0.05300 (9)	0.0216 (4)	
H44	0.9854	0.7894	0.0158	0.026*	
C45	0.95532 (15)	0.68305 (14)	0.06220 (10)	0.0242 (4)	
H45	0.9592	0.6447	0.0306	0.029*	
C46	0.93584 (15)	0.65182 (13)	0.11749 (10)	0.0237 (4)	
C47	0.91919 (15)	0.71165 (15)	0.16107 (9)	0.0238 (4)	
H47	0.8993	0.6924	0.1974	0.029*	
C48	0.93139 (15)	0.79983 (13)	0.15218 (9)	0.0222 (4)	
H48	0.9209	0.8387	0.1826	0.027*	
C49	0.97587 (17)	0.92237 (14)	0.08612 (11)	0.0280 (4)	
H49	0.9320	0.9368	0.0480	0.034*	
C50	0.9430 (2)	0.98132 (16)	0.13302 (13)	0.0370 (5)	
H50A	0.9568	1.0400	0.1226	0.056*	
H50B	0.9836	0.9678	0.1712	0.056*	
H50C	0.8673	0.9742	0.1355	0.056*	
C51	1.0920 (2)	0.93560 (18)	0.07767 (16)	0.0449 (7)	
H51A	1.1041	0.9955	0.0697	0.067*	
H51B	1.1087	0.9014	0.0444	0.067*	
H51C	1.1374	0.9185	0.1135	0.067*	
C52	0.94781 (18)	0.55922 (15)	0.13268 (14)	0.0362 (6)	
H52A	1.0224	0.5466	0.1457	0.054*	
H52B	0.9231	0.5252	0.0978	0.054*	
H52C	0.9057	0.5457	0.1644	0.054*	
Ru2	0.192005 (10)	0.281768 (9)	0.394160 (6)	0.01398 (3)	
Fe2	0.64952 (2)	0.149617 (18)	0.466743 (13)	0.01852 (5)	
Cl2	0.26782 (3)	0.40861 (3)	0.437730 (19)	0.01775 (8)	
P3	0.25353 (4)	0.22957 (3)	0.49014 (2)	0.01583 (8)	
P4	0.35009 (3)	0.27850 (3)	0.349656 (19)	0.01408 (8)	
C53	0.50097 (14)	0.18097 (12)	0.49029 (8)	0.0181 (3)	
C54	0.50307 (14)	0.19703 (12)	0.42855 (8)	0.0171 (3)	
C55	0.52886 (15)	0.11898 (12)	0.40202 (9)	0.0200 (3)	
H55	0.5358	0.1109	0.3616	0.024*	
C56	0.54245 (16)	0.05534 (13)	0.44655 (10)	0.0233 (4)	
H56	0.5590	−0.0024	0.4409	0.028*	
C57	0.52676 (15)	0.09376 (13)	0.50096 (9)	0.0218 (4)	
H57	0.5325	0.0662	0.5381	0.026*	
C58	0.77455 (17)	0.21509 (17)	0.51290 (12)	0.0327 (5)	
H58	0.7693	0.2605	0.5395	0.039*	
C59	0.77932 (16)	0.22260 (15)	0.45159 (12)	0.0308 (5)	
H59	0.7782	0.2740	0.4300	0.037*	
C60	0.78606 (16)	0.14034 (17)	0.42823 (10)	0.0295 (4)	
H60	0.7895	0.1267	0.3881	0.035*	
C61	0.78686 (17)	0.08185 (15)	0.47509 (11)	0.0291 (4)	
H61	0.7918	0.0221	0.4719	0.035*	
C62	0.77906 (17)	0.12762 (16)	0.52755 (10)	0.0304 (5)	
H62	0.7772	0.1042	0.5656	0.036*	
C63	0.47362 (14)	0.24027 (13)	0.53646 (8)	0.0188 (3)	
C64	0.55297 (16)	0.26184 (15)	0.58229 (9)	0.0258 (4)	
H64	0.6221	0.2396	0.5818	0.031*	
C65	0.53392 (18)	0.31457 (16)	0.62832 (9)	0.0298 (5)	
H65	0.5890	0.3275	0.6591	0.036*	
C66	0.43403 (18)	0.34794 (15)	0.62885 (9)	0.0271 (4)	
H66	0.4208	0.3860	0.6591	0.033*	
C67	0.35257 (16)	0.32578 (13)	0.58493 (8)	0.0217 (4)	
H67	0.2838	0.3483	0.5863	0.026*	
C68	0.36958 (14)	0.27104 (12)	0.53879 (8)	0.0178 (3)	
C69	0.48125 (13)	0.28060 (12)	0.39727 (7)	0.0151 (3)	
H69	0.4778	0.3251	0.4280	0.018*	
C70	0.57083 (14)	0.30471 (12)	0.36136 (8)	0.0182 (3)	
H70A	0.6330	0.3226	0.3882	0.027*	
H70B	0.5474	0.3512	0.3345	0.027*	
H70C	0.5891	0.2557	0.3385	0.027*	
C71	0.14538 (14)	0.25416 (14)	0.53417 (8)	0.0203 (3)	
C72	0.10979 (16)	0.33777 (14)	0.53558 (9)	0.0228 (4)	
H72	0.1413	0.3800	0.5140	0.027*	
C73	0.02884 (17)	0.36019 (16)	0.56821 (9)	0.0272 (4)	
H73	0.0056	0.4173	0.5686	0.033*	
C74	−0.01790 (17)	0.29930 (18)	0.60013 (9)	0.0319 (5)	
H74	−0.0722	0.3144	0.6230	0.038*	
C75	0.01559 (18)	0.21687 (18)	0.59812 (10)	0.0318 (5)	
H75	−0.0166	0.1749	0.6196	0.038*	
C76	0.09583 (16)	0.19330 (16)	0.56523 (9)	0.0263 (4)	
H76	0.1167	0.1357	0.5640	0.032*	
C77	0.27406 (15)	0.11599 (12)	0.50045 (8)	0.0190 (3)	
C78	0.30219 (16)	0.08134 (14)	0.55670 (9)	0.0231 (4)	
H78	0.3075	0.1168	0.5905	0.028*	
C79	0.32226 (18)	−0.00430 (14)	0.56307 (9)	0.0261 (4)	
H79	0.3403	−0.0275	0.6013	0.031*	
C80	0.31618 (19)	−0.05715 (14)	0.51368 (10)	0.0280 (4)	
H80	0.3316	−0.1157	0.5183	0.034*	
C81	0.28745 (19)	−0.02353 (14)	0.45794 (10)	0.0280 (4)	
H81	0.2820	−0.0590	0.4242	0.034*	
C82	0.26660 (17)	0.06297 (13)	0.45183 (9)	0.0227 (4)	
H82	0.2469	0.0859	0.4136	0.027*	
C83	0.35019 (14)	0.37442 (12)	0.30466 (8)	0.0171 (3)	
C84	0.30651 (15)	0.37412 (13)	0.24521 (8)	0.0205 (4)	
H84	0.2827	0.3225	0.2269	0.025*	
C85	0.29780 (17)	0.44895 (14)	0.21291 (9)	0.0261 (4)	
H85	0.2689	0.4478	0.1725	0.031*	
C86	0.33105 (17)	0.52560 (14)	0.23914 (10)	0.0274 (4)	
H86	0.3241	0.5766	0.2170	0.033*	
C87	0.37455 (17)	0.52646 (13)	0.29798 (10)	0.0248 (4)	
H87	0.3967	0.5785	0.3163	0.030*	
C88	0.38583 (15)	0.45187 (12)	0.33016 (9)	0.0196 (3)	
H88	0.4181	0.4532	0.3700	0.024*	
C89	0.36863 (14)	0.19447 (12)	0.29692 (8)	0.0174 (3)	
C90	0.32164 (17)	0.11555 (13)	0.30220 (9)	0.0217 (4)	
H90	0.2709	0.1086	0.3288	0.026*	
C91	0.3482 (2)	0.04679 (14)	0.26898 (10)	0.0285 (4)	
H91	0.3150	−0.0064	0.2725	0.034*	
C92	0.4232 (2)	0.05657 (15)	0.23085 (10)	0.0304 (5)	
H92	0.4438	0.0092	0.2095	0.037*	
C93	0.46852 (17)	0.13501 (15)	0.22348 (9)	0.0261 (4)	
H93	0.5186	0.1416	0.1965	0.031*	
C94	0.44036 (15)	0.20394 (13)	0.25578 (8)	0.0197 (3)	
H94	0.4700	0.2580	0.2499	0.024*	
C95	0.04962 (14)	0.36418 (13)	0.35484 (9)	0.0198 (4)	
C96	0.02228 (15)	0.31515 (15)	0.40297 (9)	0.0232 (4)	
H96	−0.0025	0.3428	0.4354	0.028*	
C97	0.03139 (15)	0.22695 (15)	0.40302 (9)	0.0247 (4)	
H97	0.0127	0.1959	0.4357	0.030*	
C98	0.06760 (16)	0.18277 (14)	0.35593 (10)	0.0247 (4)	
C99	0.10309 (15)	0.23188 (13)	0.31075 (9)	0.0204 (4)	
H99	0.1352	0.2045	0.2806	0.024*	
C100	0.09167 (14)	0.32160 (12)	0.30939 (8)	0.0179 (3)	
H100	0.1127	0.3528	0.2774	0.021*	
C101	0.03213 (16)	0.45892 (14)	0.35477 (10)	0.0238 (4)	
H101	0.0581	0.4809	0.3950	0.029*	
C102	0.0901 (2)	0.50596 (16)	0.31059 (12)	0.0351 (5)	
H102	0.0745	0.5665	0.3123	0.053*	
H103	0.0668	0.4846	0.2708	0.053*	
H104	0.1665	0.4970	0.3202	0.053*	
C103	−0.08756 (18)	0.47490 (16)	0.34286 (13)	0.0360 (5)	
H105	−0.1231	0.4441	0.3719	0.054*	
H106	−0.1144	0.4552	0.3032	0.054*	
H107	−0.1015	0.5356	0.3460	0.054*	
C104	0.0557 (2)	0.08810 (15)	0.35054 (13)	0.0365 (6)	
H108	−0.0147	0.0745	0.3302	0.055*	
H109	0.0641	0.0627	0.3899	0.055*	
H110	0.1098	0.0656	0.3280	0.055*	
P5	0.78723 (4)	0.23805 (3)	0.24796 (2)	0.02278 (10)	
F1	0.74472 (12)	0.19530 (10)	0.18623 (6)	0.0385 (3)	
F2	0.82868 (12)	0.27908 (11)	0.31059 (7)	0.0426 (4)	
F3	0.68177 (10)	0.29490 (9)	0.24489 (5)	0.0269 (3)	
F4	0.89232 (12)	0.18155 (10)	0.25110 (8)	0.0439 (4)	
F5	0.84234 (14)	0.31176 (10)	0.21500 (9)	0.0506 (5)	
F6	0.72950 (13)	0.16546 (9)	0.28091 (7)	0.0396 (3)	
P6	0.18582 (4)	0.76873 (5)	0.26200 (2)	0.03142 (14)	
F7	0.06412 (12)	0.78383 (15)	0.26925 (7)	0.0490 (4)	
F8	0.30486 (13)	0.74663 (16)	0.25470 (8)	0.0575 (5)	
F9	0.17071 (17)	0.82167 (15)	0.20276 (8)	0.0607 (5)	
F10	0.19791 (15)	0.71199 (13)	0.32110 (7)	0.0511 (4)	
F11	0.21866 (15)	0.84939 (13)	0.30162 (9)	0.0527 (4)	
F12	0.14987 (15)	0.68459 (14)	0.22337 (8)	0.0559 (5)	

### Atomic displacement parameters (Å^2^)

**Table:**

	*U*^11^	*U*^22^	*U*^33^	*U*^12^	*U*^13^	*U*^23^
Ru1	0.00990 (5)	0.01379 (6)	0.01686 (6)	−0.00028 (5)	0.00174 (4)	0.00289 (5)
Fe1	0.01079 (11)	0.01490 (11)	0.01875 (12)	−0.00096 (9)	0.00065 (9)	0.00157 (9)
Cl1	0.01662 (18)	0.01380 (17)	0.02113 (19)	0.00041 (14)	0.00126 (15)	0.00273 (14)
P1	0.01379 (19)	0.01402 (19)	0.01584 (19)	0.00077 (15)	0.00405 (15)	0.00159 (15)
P2	0.01156 (17)	0.01326 (19)	0.01423 (18)	−0.00034 (15)	0.00092 (14)	0.00077 (15)
C1	0.0098 (7)	0.0166 (8)	0.0181 (8)	0.0005 (6)	0.0015 (6)	0.0010 (6)
C2	0.0107 (7)	0.0153 (7)	0.0170 (8)	0.0011 (6)	0.0005 (6)	0.0015 (6)
C3	0.0125 (7)	0.0185 (8)	0.0195 (8)	−0.0009 (6)	0.0014 (6)	0.0043 (6)
C4	0.0151 (8)	0.0150 (8)	0.0301 (10)	−0.0013 (6)	0.0015 (7)	0.0026 (7)
C5	0.0149 (8)	0.0159 (8)	0.0224 (8)	0.0014 (6)	0.0002 (6)	−0.0018 (6)
C6	0.0151 (8)	0.0268 (10)	0.0424 (12)	0.0034 (7)	0.0054 (8)	0.0111 (9)
C7	0.0111 (8)	0.0415 (12)	0.0372 (12)	−0.0014 (8)	0.0038 (8)	−0.0131 (10)
C8	0.0154 (9)	0.0537 (15)	0.0351 (12)	0.0021 (9)	0.0060 (8)	0.0214 (11)
C9	0.0145 (9)	0.0197 (10)	0.0781 (19)	−0.0042 (7)	0.0049 (10)	−0.0047 (11)
C10	0.0128 (8)	0.0596 (16)	0.0262 (10)	0.0035 (9)	−0.0029 (7)	−0.0116 (10)
C11	0.0177 (8)	0.0139 (7)	0.0123 (7)	0.0005 (6)	0.0010 (6)	−0.0008 (6)
C12	0.0168 (8)	0.0241 (9)	0.0161 (7)	0.0024 (7)	0.0016 (6)	0.0019 (7)
C13	0.0225 (9)	0.0226 (9)	0.0155 (8)	0.0051 (7)	−0.0015 (7)	0.0007 (7)
C14	0.0274 (9)	0.0181 (8)	0.0151 (8)	0.0013 (7)	0.0022 (7)	0.0016 (6)
C15	0.0221 (9)	0.0156 (8)	0.0203 (8)	−0.0013 (6)	0.0046 (7)	0.0016 (6)
C16	0.0159 (7)	0.0136 (7)	0.0146 (7)	0.0012 (6)	0.0018 (5)	−0.0013 (6)
C17	0.0109 (6)	0.0151 (7)	0.0154 (7)	−0.0003 (6)	0.0024 (5)	0.0004 (6)
C18	0.0145 (7)	0.0221 (8)	0.0190 (8)	0.0009 (7)	0.0050 (6)	−0.0005 (7)
C19	0.0178 (8)	0.0230 (9)	0.0202 (8)	0.0022 (7)	0.0060 (6)	0.0049 (7)
C20	0.0224 (9)	0.0287 (10)	0.0237 (9)	−0.0037 (8)	0.0058 (7)	0.0056 (8)
C21	0.0231 (10)	0.0407 (13)	0.0319 (11)	−0.0071 (9)	0.0065 (8)	0.0134 (9)
C22	0.0269 (10)	0.0548 (16)	0.0272 (10)	0.0085 (10)	0.0131 (8)	0.0180 (10)
C23	0.0374 (12)	0.0457 (14)	0.0267 (11)	0.0175 (11)	0.0173 (9)	0.0105 (10)
C24	0.0332 (11)	0.0264 (10)	0.0221 (9)	0.0077 (8)	0.0094 (8)	0.0059 (8)
C25	0.0152 (7)	0.0146 (8)	0.0206 (8)	0.0004 (6)	0.0050 (6)	−0.0008 (6)
C26	0.0238 (9)	0.0214 (9)	0.0239 (9)	0.0027 (7)	−0.0026 (7)	0.0000 (7)
C27	0.0227 (9)	0.0208 (9)	0.0276 (10)	0.0002 (7)	−0.0044 (8)	−0.0050 (7)
C28	0.0251 (9)	0.0172 (9)	0.0298 (10)	−0.0021 (7)	0.0065 (8)	−0.0028 (7)
C29	0.0349 (11)	0.0168 (8)	0.0209 (9)	−0.0006 (8)	0.0070 (8)	0.0027 (7)
C30	0.0261 (9)	0.0172 (8)	0.0159 (8)	0.0003 (7)	0.0060 (7)	−0.0003 (6)
C31	0.0154 (8)	0.0183 (8)	0.0190 (8)	−0.0007 (6)	0.0022 (6)	−0.0019 (6)
C32	0.0204 (8)	0.0228 (9)	0.0209 (9)	−0.0006 (7)	−0.0014 (7)	−0.0015 (7)
C33	0.0266 (10)	0.0308 (11)	0.0282 (10)	−0.0026 (8)	−0.0035 (8)	−0.0095 (9)
C34	0.0291 (11)	0.0275 (11)	0.0349 (12)	−0.0031 (8)	0.0015 (9)	−0.0140 (9)
C35	0.0258 (10)	0.0191 (9)	0.0378 (12)	0.0011 (7)	0.0057 (9)	−0.0063 (8)
C36	0.0179 (8)	0.0189 (8)	0.0225 (9)	0.0008 (7)	0.0025 (7)	−0.0021 (7)
C37	0.0124 (7)	0.0172 (8)	0.0147 (7)	−0.0009 (6)	−0.0005 (6)	0.0033 (6)
C38	0.0176 (8)	0.0174 (8)	0.0170 (8)	−0.0007 (6)	0.0009 (6)	0.0020 (6)
C39	0.0241 (9)	0.0168 (8)	0.0264 (9)	−0.0022 (7)	0.0003 (7)	0.0041 (7)
C40	0.0256 (9)	0.0260 (10)	0.0232 (9)	−0.0061 (8)	−0.0012 (7)	0.0115 (8)
C41	0.0237 (9)	0.0337 (11)	0.0153 (8)	−0.0056 (8)	0.0026 (7)	0.0057 (7)
C42	0.0192 (8)	0.0212 (9)	0.0167 (8)	−0.0008 (7)	0.0018 (6)	0.0011 (6)
C43	0.0115 (8)	0.0218 (9)	0.0291 (10)	−0.0032 (6)	−0.0002 (7)	0.0039 (7)
C44	0.0112 (7)	0.0267 (10)	0.0275 (9)	−0.0014 (6)	0.0045 (6)	0.0054 (7)
C45	0.0127 (8)	0.0238 (9)	0.0359 (11)	0.0039 (7)	0.0028 (7)	0.0014 (8)
C46	0.0117 (8)	0.0218 (9)	0.0367 (11)	0.0018 (7)	−0.0006 (7)	0.0089 (8)
C47	0.0122 (8)	0.0340 (11)	0.0240 (9)	−0.0013 (7)	−0.0024 (7)	0.0105 (8)
C48	0.0139 (8)	0.0279 (10)	0.0234 (9)	−0.0040 (7)	−0.0025 (7)	−0.0015 (7)
C49	0.0202 (9)	0.0214 (9)	0.0414 (12)	−0.0070 (7)	0.0005 (8)	0.0051 (8)
C50	0.0316 (12)	0.0262 (11)	0.0521 (15)	−0.0052 (9)	0.0009 (11)	−0.0059 (10)
C51	0.0278 (12)	0.0319 (13)	0.078 (2)	−0.0114 (10)	0.0166 (13)	0.0040 (13)
C52	0.0193 (10)	0.0238 (11)	0.0647 (17)	0.0055 (8)	0.0019 (10)	0.0159 (11)
Ru2	0.01221 (6)	0.01695 (6)	0.01265 (6)	−0.00117 (5)	0.00112 (4)	0.00074 (5)
Fe2	0.01441 (11)	0.01891 (12)	0.02201 (13)	0.00173 (9)	0.00146 (9)	0.00385 (10)
Cl2	0.01743 (18)	0.01844 (19)	0.01753 (18)	−0.00152 (15)	0.00287 (14)	−0.00190 (14)
P3	0.01478 (19)	0.0194 (2)	0.01334 (19)	−0.00159 (15)	0.00181 (15)	0.00164 (15)
P4	0.01429 (18)	0.01534 (18)	0.01277 (17)	0.00003 (16)	0.00234 (14)	0.00005 (16)
C53	0.0147 (8)	0.0213 (8)	0.0180 (8)	0.0001 (6)	0.0009 (6)	0.0024 (6)
C54	0.0161 (8)	0.0184 (8)	0.0166 (8)	0.0003 (6)	0.0014 (6)	0.0015 (6)
C55	0.0180 (8)	0.0199 (9)	0.0219 (9)	0.0011 (7)	0.0023 (7)	−0.0014 (7)
C56	0.0209 (9)	0.0184 (9)	0.0302 (10)	0.0001 (7)	0.0024 (7)	0.0029 (7)
C57	0.0185 (8)	0.0219 (9)	0.0248 (9)	0.0002 (7)	0.0023 (7)	0.0052 (7)
C58	0.0167 (9)	0.0369 (12)	0.0430 (13)	−0.0005 (8)	−0.0014 (9)	−0.0035 (10)
C59	0.0150 (9)	0.0286 (10)	0.0481 (13)	0.0007 (7)	0.0014 (8)	0.0129 (9)
C60	0.0179 (9)	0.0401 (12)	0.0315 (11)	0.0030 (8)	0.0067 (8)	0.0048 (9)
C61	0.0188 (9)	0.0261 (10)	0.0424 (12)	0.0069 (8)	0.0042 (8)	0.0067 (9)
C62	0.0180 (9)	0.0414 (13)	0.0306 (11)	0.0018 (8)	−0.0013 (8)	0.0093 (9)
C63	0.0171 (8)	0.0234 (9)	0.0155 (7)	−0.0005 (7)	0.0008 (6)	0.0023 (7)
C64	0.0188 (8)	0.0377 (12)	0.0196 (8)	−0.0013 (8)	−0.0024 (7)	0.0006 (8)
C65	0.0254 (10)	0.0423 (12)	0.0198 (9)	−0.0047 (9)	−0.0044 (8)	−0.0039 (9)
C66	0.0280 (10)	0.0337 (11)	0.0191 (9)	−0.0029 (8)	0.0012 (8)	−0.0054 (8)
C67	0.0215 (9)	0.0260 (10)	0.0173 (8)	0.0001 (7)	0.0015 (7)	−0.0018 (7)
C68	0.0172 (8)	0.0222 (9)	0.0136 (7)	−0.0014 (6)	0.0004 (6)	0.0023 (6)
C69	0.0142 (7)	0.0167 (7)	0.0143 (7)	0.0008 (6)	0.0014 (5)	0.0003 (6)
C70	0.0149 (8)	0.0210 (8)	0.0191 (8)	0.0000 (6)	0.0040 (6)	0.0024 (6)
C71	0.0169 (8)	0.0288 (9)	0.0153 (7)	−0.0020 (7)	0.0019 (6)	0.0012 (7)
C72	0.0199 (9)	0.0315 (10)	0.0174 (8)	−0.0019 (7)	0.0041 (7)	−0.0029 (7)
C73	0.0209 (9)	0.0399 (12)	0.0213 (9)	0.0010 (8)	0.0052 (7)	−0.0041 (8)
C74	0.0207 (9)	0.0562 (16)	0.0198 (9)	−0.0016 (9)	0.0069 (7)	−0.0006 (9)
C75	0.0238 (10)	0.0497 (14)	0.0228 (10)	−0.0049 (10)	0.0061 (8)	0.0084 (9)
C76	0.0205 (9)	0.0368 (11)	0.0217 (9)	−0.0041 (8)	0.0036 (7)	0.0058 (8)
C77	0.0171 (8)	0.0212 (8)	0.0186 (8)	−0.0037 (6)	0.0020 (6)	0.0022 (6)
C78	0.0256 (9)	0.0255 (10)	0.0176 (8)	−0.0019 (7)	0.0008 (7)	0.0023 (7)
C79	0.0319 (10)	0.0241 (10)	0.0220 (9)	−0.0016 (8)	0.0026 (8)	0.0086 (7)
C80	0.0329 (11)	0.0203 (9)	0.0306 (11)	−0.0022 (8)	0.0034 (9)	0.0053 (8)
C81	0.0373 (12)	0.0232 (10)	0.0231 (10)	−0.0015 (8)	0.0020 (8)	−0.0009 (8)
C82	0.0266 (10)	0.0238 (9)	0.0171 (8)	−0.0007 (7)	0.0014 (7)	0.0028 (7)
C83	0.0154 (8)	0.0204 (8)	0.0162 (8)	0.0034 (6)	0.0053 (6)	0.0031 (6)
C84	0.0199 (8)	0.0246 (9)	0.0171 (8)	0.0045 (7)	0.0032 (7)	0.0028 (7)
C85	0.0266 (10)	0.0312 (11)	0.0207 (9)	0.0087 (8)	0.0042 (7)	0.0067 (8)
C86	0.0262 (10)	0.0264 (10)	0.0315 (11)	0.0069 (8)	0.0111 (8)	0.0123 (8)
C87	0.0247 (9)	0.0196 (9)	0.0316 (10)	0.0012 (7)	0.0092 (8)	0.0040 (8)
C88	0.0169 (8)	0.0203 (8)	0.0224 (9)	0.0007 (6)	0.0055 (7)	0.0016 (7)
C89	0.0176 (8)	0.0187 (8)	0.0155 (8)	0.0011 (6)	0.0004 (6)	−0.0013 (6)
C90	0.0267 (9)	0.0201 (9)	0.0189 (8)	−0.0012 (7)	0.0052 (7)	−0.0006 (7)
C91	0.0381 (12)	0.0202 (9)	0.0274 (10)	−0.0022 (8)	0.0051 (9)	−0.0053 (8)
C92	0.0376 (12)	0.0277 (11)	0.0266 (10)	0.0053 (9)	0.0065 (9)	−0.0077 (8)
C93	0.0282 (10)	0.0302 (10)	0.0205 (9)	0.0050 (8)	0.0056 (7)	−0.0044 (8)
C94	0.0194 (8)	0.0227 (9)	0.0170 (8)	0.0015 (7)	0.0024 (6)	−0.0003 (7)
C95	0.0123 (8)	0.0257 (9)	0.0203 (9)	0.0021 (7)	−0.0021 (6)	−0.0009 (7)
C96	0.0139 (8)	0.0371 (11)	0.0187 (8)	0.0005 (7)	0.0019 (6)	0.0007 (8)
C97	0.0142 (8)	0.0365 (11)	0.0224 (9)	−0.0071 (7)	−0.0015 (7)	0.0070 (8)
C98	0.0160 (8)	0.0244 (10)	0.0310 (10)	−0.0058 (7)	−0.0069 (7)	0.0026 (8)
C99	0.0174 (8)	0.0226 (9)	0.0192 (8)	0.0011 (7)	−0.0047 (6)	−0.0035 (7)
C100	0.0157 (8)	0.0209 (9)	0.0160 (8)	0.0019 (6)	−0.0015 (6)	0.0002 (6)
C101	0.0192 (9)	0.0238 (9)	0.0279 (10)	0.0052 (7)	0.0010 (7)	−0.0045 (8)
C102	0.0365 (12)	0.0245 (10)	0.0466 (14)	0.0058 (9)	0.0142 (11)	0.0050 (10)
C103	0.0193 (10)	0.0311 (12)	0.0567 (16)	0.0083 (8)	0.0008 (10)	−0.0061 (11)
C104	0.0288 (11)	0.0236 (11)	0.0528 (15)	−0.0085 (9)	−0.0115 (10)	0.0038 (10)
P5	0.0201 (2)	0.0208 (2)	0.0265 (2)	0.00140 (18)	−0.00101 (18)	0.00080 (19)
F1	0.0438 (8)	0.0446 (8)	0.0253 (7)	0.0173 (7)	−0.0023 (6)	−0.0078 (6)
F2	0.0393 (8)	0.0397 (8)	0.0435 (8)	−0.0025 (7)	−0.0144 (6)	−0.0128 (7)
F3	0.0245 (6)	0.0316 (7)	0.0255 (6)	0.0070 (5)	0.0068 (5)	0.0020 (5)
F4	0.0279 (7)	0.0374 (8)	0.0621 (10)	0.0148 (6)	−0.0099 (7)	−0.0082 (7)
F5	0.0466 (9)	0.0327 (8)	0.0820 (13)	0.0024 (7)	0.0438 (9)	0.0097 (8)
F6	0.0550 (9)	0.0250 (7)	0.0391 (8)	−0.0076 (6)	0.0073 (7)	0.0051 (6)
P6	0.0213 (2)	0.0539 (4)	0.0191 (2)	0.0029 (2)	0.00287 (18)	−0.0044 (2)
F7	0.0253 (7)	0.0901 (13)	0.0308 (7)	0.0037 (8)	0.0008 (5)	−0.0144 (9)
F8	0.0283 (7)	0.0988 (16)	0.0470 (9)	0.0043 (9)	0.0110 (7)	−0.0142 (11)
F9	0.0672 (12)	0.0799 (15)	0.0352 (9)	−0.0051 (11)	0.0072 (8)	0.0177 (9)
F10	0.0538 (10)	0.0666 (12)	0.0320 (8)	0.0066 (9)	0.0018 (7)	0.0066 (8)
F11	0.0493 (10)	0.0511 (10)	0.0542 (11)	−0.0089 (8)	−0.0065 (8)	−0.0161 (9)
F12	0.0555 (11)	0.0645 (12)	0.0449 (10)	0.0054 (9)	−0.0047 (8)	−0.0222 (9)

### Geometric parameters (Å, °)

**Table:**

Ru1—P1	2.3896 (5)	Ru2—C98	2.318 (2)
Ru1—P2	2.3810 (5)	Ru2—C99	2.235 (2)
Ru1—Cl1	2.3967 (4)	Ru2—C100	2.272 (2)
Ru1—C43	2.301 (2)	Fe2—C53	2.094 (2)
Ru1—C44	2.262 (2)	Fe2—C54	2.096 (2)
Ru1—C45	2.253 (2)	Fe2—C55	2.052 (2)
Ru1—C46	2.329 (2)	Fe2—C56	2.034 (2)
Ru1—C47	2.250 (2)	Fe2—C57	2.039 (2)
Ru1—C48	2.257 (2)	Fe2—C58	2.072 (2)
Fe1—C1	2.093 (2)	Fe2—C59	2.083 (2)
Fe1—C2	2.099 (2)	Fe2—C60	2.055 (2)
Fe1—C3	2.050 (2)	Fe2—C61	2.040 (2)
Fe1—C4	2.039 (2)	Fe2—C62	2.048 (2)
Fe1—C5	2.038 (2)	P3—C77	1.826 (2)
Fe1—C6	2.077 (2)	P3—C71	1.8504 (19)
Fe1—C7	2.084 (2)	P3—C68	1.8534 (19)
Fe1—C8	2.058 (2)	P4—C89	1.8305 (19)
Fe1—C9	2.030 (2)	P4—C83	1.8331 (19)
Fe1—C10	2.048 (2)	P4—C69	1.8753 (17)
P1—C25	1.8201 (19)	C53—C57	1.431 (3)
P1—C19	1.8454 (19)	C53—C54	1.439 (3)
P1—C16	1.8456 (18)	C53—C63	1.487 (3)
P2—C37	1.8261 (18)	C54—C55	1.432 (3)
P2—C31	1.8299 (19)	C54—C69	1.512 (3)
P2—C17	1.8767 (17)	C55—C56	1.427 (3)
C1—C5	1.432 (3)	C55—H55	0.9500
C1—C2	1.444 (2)	C56—C57	1.423 (3)
C1—C11	1.486 (2)	C56—H56	0.9500
C2—C3	1.438 (2)	C57—H57	0.9500
C2—C17	1.512 (2)	C58—C59	1.417 (4)
C3—C4	1.426 (3)	C58—C62	1.422 (4)
C3—H3	0.9500	C58—H58	0.9500
C4—C5	1.419 (3)	C59—C60	1.413 (4)
C4—H4	0.9500	C59—H59	0.9500
C5—H5	0.9500	C60—C61	1.415 (3)
C6—C7	1.396 (3)	C60—H60	0.9500
C6—C10	1.414 (4)	C61—C62	1.416 (4)
C6—H6	0.9500	C61—H61	0.9500
C7—C8	1.393 (4)	C62—H62	0.9500
C7—H7	0.9500	C63—C64	1.403 (3)
C8—C9	1.415 (4)	C63—C68	1.420 (3)
C8—H8	0.9500	C64—C65	1.389 (3)
C9—C10	1.420 (4)	C64—H64	0.9500
C9—H9	0.9500	C65—C66	1.380 (3)
C10—H10	0.9500	C65—H65	0.9500
C11—C12	1.400 (2)	C66—C67	1.394 (3)
C11—C16	1.415 (2)	C66—H66	0.9500
C12—C13	1.381 (3)	C67—C68	1.404 (3)
C12—H12	0.9500	C67—H67	0.9500
C13—C14	1.387 (3)	C69—C70	1.538 (2)
C13—H13	0.9500	C69—H69	1.0000
C14—C15	1.382 (3)	C70—H70A	0.9800
C14—H14	0.9500	C70—H70B	0.9800
C15—C16	1.405 (2)	C70—H70C	0.9800
C15—H15	0.9500	C71—C76	1.395 (3)
C17—C18	1.543 (2)	C71—C72	1.399 (3)
C17—H17	1.0000	C72—C73	1.395 (3)
C18—H18A	0.9800	C72—H72	0.9500
C18—H18B	0.9800	C73—C74	1.388 (3)
C18—H18C	0.9800	C73—H73	0.9500
C19—C24	1.394 (3)	C74—C75	1.374 (4)
C19—C20	1.408 (3)	C74—H74	0.9500
C20—C21	1.396 (3)	C75—C76	1.396 (3)
C20—H20	0.9500	C75—H75	0.9500
C21—C22	1.388 (4)	C76—H76	0.9500
C21—H21	0.9500	C77—C82	1.387 (3)
C22—C23	1.380 (4)	C77—C78	1.403 (3)
C22—H22	0.9500	C78—C79	1.382 (3)
C23—C24	1.406 (3)	C78—H78	0.9500
C23—H23	0.9500	C79—C80	1.400 (3)
C24—H24	0.9500	C79—H79	0.9500
C25—C30	1.386 (3)	C80—C81	1.387 (3)
C25—C26	1.402 (3)	C80—H80	0.9500
C26—C27	1.386 (3)	C81—C82	1.397 (3)
C26—H26	0.9500	C81—H81	0.9500
C27—C28	1.384 (3)	C82—H82	0.9500
C27—H27	0.9500	C83—C84	1.403 (3)
C28—C29	1.387 (3)	C83—C88	1.407 (3)
C28—H28	0.9500	C84—C85	1.392 (3)
C29—C30	1.388 (3)	C84—H84	0.9500
C29—H29	0.9500	C85—C86	1.394 (3)
C30—H30	0.9500	C85—H85	0.9500
C31—C32	1.403 (3)	C86—C87	1.389 (3)
C31—C36	1.405 (3)	C86—H86	0.9500
C32—C33	1.393 (3)	C87—C88	1.388 (3)
C32—H32	0.9500	C87—H87	0.9500
C33—C34	1.388 (3)	C88—H88	0.9500
C33—H33	0.9500	C89—C90	1.396 (3)
C34—C35	1.394 (3)	C89—C94	1.402 (3)
C34—H34	0.9500	C90—C91	1.393 (3)
C35—C36	1.393 (3)	C90—H90	0.9500
C35—H35	0.9500	C91—C92	1.384 (3)
C36—H36	0.9500	C91—H91	0.9500
C37—C38	1.391 (3)	C92—C93	1.387 (3)
C37—C42	1.400 (3)	C92—H92	0.9500
C38—C39	1.392 (3)	C93—C94	1.390 (3)
C38—H38	0.9500	C93—H93	0.9500
C39—C40	1.386 (3)	C94—H94	0.9500
C39—H39	0.9500	C95—C100	1.402 (3)
C40—C41	1.388 (3)	C95—C96	1.426 (3)
C40—H40	0.9500	C95—C101	1.514 (3)
C41—C42	1.390 (3)	C96—C97	1.399 (3)
C41—H41	0.9500	C96—H96	0.9500
C42—H42	0.9500	C97—C98	1.411 (3)
C43—C44	1.408 (3)	C97—H97	0.9500
C43—C48	1.420 (3)	C98—C99	1.413 (3)
C43—C49	1.508 (3)	C98—C104	1.508 (3)
C44—C45	1.410 (3)	C99—C100	1.426 (3)
C44—H44	0.9500	C99—H99	0.9500
C45—C46	1.410 (3)	C100—H100	0.9500
C45—H45	0.9500	C101—C102	1.521 (3)
C46—C47	1.410 (3)	C101—C103	1.536 (3)
C46—C52	1.508 (3)	C101—H101	1.0000
C47—C48	1.420 (3)	C102—H102	0.9800
C47—H47	0.9500	C102—H103	0.9800
C48—H48	0.9500	C102—H104	0.9800
C49—C50	1.521 (4)	C103—H105	0.9800
C49—C51	1.532 (3)	C103—H106	0.9800
C49—H49	1.0000	C103—H107	0.9800
C50—H50A	0.9800	C104—H108	0.9800
C50—H50B	0.9800	C104—H109	0.9800
C50—H50C	0.9800	C104—H110	0.9800
C51—H51A	0.9800	P5—F1	1.5969 (15)
C51—H51B	0.9800	P5—F5	1.5991 (16)
C51—H51C	0.9800	P5—F2	1.6003 (16)
C52—H52A	0.9800	P5—F6	1.6031 (16)
C52—H52B	0.9800	P5—F4	1.6041 (15)
C52—H52C	0.9800	P5—F3	1.6111 (13)
Ru2—P3	2.3834 (5)	P6—F9	1.5840 (19)
Ru2—P4	2.3725 (5)	P6—F8	1.5868 (17)
Ru2—Cl2	2.3880 (5)	P6—F11	1.5894 (19)
Ru2—C95	2.320 (2)	P6—F7	1.5992 (16)
Ru2—C96	2.261 (2)	P6—F10	1.6141 (18)
Ru2—C97	2.256 (2)	P6—F12	1.630 (2)
			
C47—Ru1—C45	64.71 (8)	C99—Ru2—P4	89.49 (5)
C47—Ru1—C48	36.74 (8)	C97—Ru2—P4	149.41 (6)
C45—Ru1—C48	77.14 (8)	C96—Ru2—P4	156.73 (5)
C47—Ru1—C44	76.43 (7)	C100—Ru2—P4	93.18 (5)
C45—Ru1—C44	36.40 (7)	C98—Ru2—P4	113.63 (6)
C48—Ru1—C44	64.93 (7)	C95—Ru2—P4	120.74 (5)
C47—Ru1—C43	65.34 (7)	C99—Ru2—P3	137.39 (5)
C45—Ru1—C43	65.28 (8)	C97—Ru2—P3	88.80 (5)
C48—Ru1—C43	36.28 (7)	C96—Ru2—P3	101.77 (5)
C44—Ru1—C43	35.92 (7)	C100—Ru2—P3	165.00 (5)
C47—Ru1—C46	35.82 (8)	C98—Ru2—P3	104.03 (6)
C45—Ru1—C46	35.80 (8)	C95—Ru2—P3	134.46 (5)
C48—Ru1—C46	65.27 (8)	P4—Ru2—P3	101.020 (16)
C44—Ru1—C46	64.66 (7)	C99—Ru2—Cl2	141.88 (5)
C43—Ru1—C46	76.63 (7)	C97—Ru2—Cl2	127.46 (6)
C47—Ru1—P2	86.73 (5)	C96—Ru2—Cl2	96.16 (6)
C45—Ru1—P2	141.85 (6)	C100—Ru2—Cl2	106.10 (5)
C48—Ru1—P2	95.25 (5)	C98—Ru2—Cl2	160.94 (6)
C44—Ru1—P2	160.13 (5)	C95—Ru2—Cl2	86.85 (5)
C43—Ru1—P2	126.57 (6)	P4—Ru2—Cl2	82.939 (16)
C46—Ru1—P2	106.96 (5)	P3—Ru2—Cl2	80.684 (17)
C47—Ru1—P1	144.84 (6)	C56—Fe2—C57	40.90 (8)
C45—Ru1—P1	89.68 (6)	C56—Fe2—C61	100.33 (9)
C48—Ru1—P1	162.35 (5)	C57—Fe2—C61	115.36 (9)
C44—Ru1—P1	97.51 (5)	C56—Fe2—C62	119.28 (9)
C43—Ru1—P1	127.09 (5)	C57—Fe2—C62	104.89 (9)
C46—Ru1—P1	110.07 (6)	C61—Fe2—C62	40.54 (10)
P2—Ru1—P1	102.339 (16)	C56—Fe2—C55	40.87 (8)
C47—Ru1—Cl1	134.70 (6)	C57—Fe2—C55	68.53 (8)
C45—Ru1—Cl1	134.76 (6)	C61—Fe2—C55	119.89 (9)
C48—Ru1—Cl1	100.48 (6)	C62—Fe2—C55	156.18 (9)
C44—Ru1—Cl1	101.05 (5)	C56—Fe2—C60	115.53 (10)
C43—Ru1—Cl1	86.05 (5)	C57—Fe2—C60	150.24 (9)
C46—Ru1—Cl1	162.68 (5)	C61—Fe2—C60	40.43 (9)
P2—Ru1—Cl1	83.256 (16)	C62—Fe2—C60	68.04 (9)
P1—Ru1—Cl1	80.405 (16)	C55—Fe2—C60	105.74 (9)
C9—Fe1—C5	112.13 (9)	C56—Fe2—C58	158.69 (10)
C9—Fe1—C4	101.30 (9)	C57—Fe2—C58	126.76 (10)
C5—Fe1—C4	40.73 (8)	C61—Fe2—C58	67.67 (10)
C9—Fe1—C10	40.74 (11)	C62—Fe2—C58	40.38 (10)
C5—Fe1—C10	105.75 (8)	C55—Fe2—C58	160.40 (9)
C4—Fe1—C10	123.94 (9)	C60—Fe2—C58	67.42 (10)
C9—Fe1—C3	124.09 (9)	C56—Fe2—C59	153.20 (10)
C5—Fe1—C3	68.53 (7)	C57—Fe2—C59	165.79 (10)
C4—Fe1—C3	40.83 (8)	C61—Fe2—C59	67.41 (9)
C10—Fe1—C3	162.05 (10)	C62—Fe2—C59	67.59 (9)
C9—Fe1—C8	40.50 (12)	C55—Fe2—C59	123.27 (9)
C5—Fe1—C8	145.76 (10)	C60—Fe2—C59	39.93 (10)
C4—Fe1—C8	113.28 (9)	C58—Fe2—C59	39.89 (10)
C10—Fe1—C8	67.61 (10)	C56—Fe2—C53	68.24 (8)
C3—Fe1—C8	106.97 (8)	C57—Fe2—C53	40.49 (8)
C9—Fe1—C6	67.46 (9)	C61—Fe2—C53	153.34 (9)
C5—Fe1—C6	131.00 (9)	C62—Fe2—C53	122.83 (9)
C4—Fe1—C6	163.87 (9)	C55—Fe2—C53	67.84 (8)
C10—Fe1—C6	40.09 (10)	C60—Fe2—C53	166.22 (8)
C3—Fe1—C6	155.17 (9)	C58—Fe2—C53	114.35 (9)
C8—Fe1—C6	66.29 (9)	C59—Fe2—C53	132.52 (9)
C9—Fe1—C7	67.25 (10)	C56—Fe2—C54	68.37 (8)
C5—Fe1—C7	170.16 (9)	C57—Fe2—C54	68.20 (8)
C4—Fe1—C7	148.74 (9)	C61—Fe2—C54	159.15 (9)
C10—Fe1—C7	67.03 (9)	C62—Fe2—C54	160.30 (9)
C3—Fe1—C7	120.41 (9)	C55—Fe2—C54	40.38 (7)
C8—Fe1—C7	39.28 (11)	C60—Fe2—C54	127.22 (8)
C6—Fe1—C7	39.21 (10)	C58—Fe2—C54	128.29 (9)
C9—Fe1—C1	148.36 (10)	C59—Fe2—C54	114.62 (8)
C5—Fe1—C1	40.55 (7)	C53—Fe2—C54	40.19 (7)
C4—Fe1—C1	68.16 (7)	C77—P3—C71	103.93 (9)
C10—Fe1—C1	119.43 (9)	C77—P3—C68	100.38 (9)
C3—Fe1—C1	68.02 (7)	C71—P3—C68	101.05 (8)
C8—Fe1—C1	171.13 (10)	C77—P3—Ru2	118.97 (6)
C6—Fe1—C1	114.96 (8)	C71—P3—Ru2	104.44 (6)
C7—Fe1—C1	136.11 (8)	C68—P3—Ru2	125.04 (6)
C9—Fe1—C2	164.33 (10)	C89—P4—C83	102.73 (8)
C5—Fe1—C2	68.29 (7)	C89—P4—C69	102.42 (8)
C4—Fe1—C2	68.37 (7)	C83—P4—C69	104.30 (9)
C10—Fe1—C2	154.90 (9)	C89—P4—Ru2	119.19 (6)
C3—Fe1—C2	40.54 (7)	C83—P4—Ru2	106.56 (6)
C8—Fe1—C2	131.26 (9)	C69—P4—Ru2	119.55 (5)
C6—Fe1—C2	124.97 (8)	C57—C53—C54	107.74 (17)
C7—Fe1—C2	115.17 (8)	C57—C53—C63	123.76 (17)
C1—Fe1—C2	40.29 (7)	C54—C53—C63	128.46 (17)
C25—P1—C19	103.11 (9)	C57—C53—Fe2	67.69 (11)
C25—P1—C16	101.92 (8)	C54—C53—Fe2	69.98 (10)
C19—P1—C16	100.80 (8)	C63—C53—Fe2	129.38 (13)
C25—P1—Ru1	118.06 (6)	C55—C54—C53	107.37 (16)
C19—P1—Ru1	106.40 (7)	C55—C54—C69	126.20 (16)
C16—P1—Ru1	123.57 (6)	C53—C54—C69	126.43 (17)
C37—P2—C31	102.06 (8)	C55—C54—Fe2	68.16 (11)
C37—P2—C17	103.01 (8)	C53—C54—Fe2	69.84 (10)
C31—P2—C17	105.86 (8)	C69—C54—Fe2	127.60 (12)
C37—P2—Ru1	118.76 (6)	C56—C55—C54	108.55 (17)
C31—P2—Ru1	104.85 (6)	C56—C55—Fe2	68.88 (11)
C17—P2—Ru1	120.24 (5)	C54—C55—Fe2	71.46 (11)
C5—C1—C2	107.73 (16)	C56—C55—H55	125.7
C5—C1—C11	123.22 (16)	C54—C55—H55	125.7
C2—C1—C11	129.05 (16)	Fe2—C55—H55	125.5
C5—C1—Fe1	67.67 (10)	C57—C56—C55	107.85 (18)
C2—C1—Fe1	70.08 (9)	C57—C56—Fe2	69.75 (12)
C11—C1—Fe1	128.26 (12)	C55—C56—Fe2	70.24 (11)
C3—C2—C1	107.06 (16)	C57—C56—H56	126.1
C3—C2—C17	126.14 (16)	C55—C56—H56	126.1
C1—C2—C17	126.76 (16)	Fe2—C56—H56	125.5
C3—C2—Fe1	67.89 (10)	C56—C57—C53	108.47 (17)
C1—C2—Fe1	69.62 (9)	C56—C57—Fe2	69.36 (12)
C17—C2—Fe1	129.27 (12)	C53—C57—Fe2	71.82 (11)
C4—C3—C2	108.57 (16)	C56—C57—H57	125.8
C4—C3—Fe1	69.20 (10)	C53—C57—H57	125.8
C2—C3—Fe1	71.57 (10)	Fe2—C57—H57	124.6
C4—C3—H3	125.7	C59—C58—C62	108.0 (2)
C2—C3—H3	125.7	C59—C58—Fe2	70.44 (13)
Fe1—C3—H3	125.1	C62—C58—Fe2	68.89 (13)
C5—C4—C3	107.97 (16)	C59—C58—H58	126.0
C5—C4—Fe1	69.58 (11)	C62—C58—H58	126.0
C3—C4—Fe1	69.97 (11)	Fe2—C58—H58	126.3
C5—C4—H4	126.0	C60—C59—C58	108.1 (2)
C3—C4—H4	126.0	C60—C59—Fe2	68.98 (13)
Fe1—C4—H4	126.0	C58—C59—Fe2	69.67 (13)
C4—C5—C1	108.64 (16)	C60—C59—H59	126.0
C4—C5—Fe1	69.69 (11)	C58—C59—H59	126.0
C1—C5—Fe1	71.78 (10)	Fe2—C59—H59	126.9
C4—C5—H5	125.7	C59—C60—C61	108.0 (2)
C1—C5—H5	125.7	C59—C60—Fe2	71.09 (13)
Fe1—C5—H5	124.4	C61—C60—Fe2	69.24 (12)
C7—C6—C10	108.6 (2)	C59—C60—H60	126.0
C7—C6—Fe1	70.68 (13)	C61—C60—H60	126.0
C10—C6—Fe1	68.85 (12)	Fe2—C60—H60	125.3
C7—C6—H6	125.7	C60—C61—C62	108.3 (2)
C10—C6—H6	125.7	C60—C61—Fe2	70.33 (12)
Fe1—C6—H6	126.3	C62—C61—Fe2	70.02 (12)
C8—C7—C6	108.3 (2)	C60—C61—H61	125.8
C8—C7—Fe1	69.34 (13)	C62—C61—H61	125.8
C6—C7—Fe1	70.11 (12)	Fe2—C61—H61	125.4
C8—C7—H7	125.8	C61—C62—C58	107.6 (2)
C6—C7—H7	125.8	C61—C62—Fe2	69.44 (12)
Fe1—C7—H7	126.3	C58—C62—Fe2	70.73 (13)
C7—C8—C9	108.5 (2)	C61—C62—H62	126.2
C7—C8—Fe1	71.37 (13)	C58—C62—H62	126.2
C9—C8—Fe1	68.68 (13)	Fe2—C62—H62	125.2
C7—C8—H8	125.8	C64—C63—C68	118.42 (18)
C9—C8—H8	125.8	C64—C63—C53	118.19 (17)
Fe1—C8—H8	125.8	C68—C63—C53	123.22 (16)
C8—C9—C10	107.4 (2)	C65—C64—C63	122.3 (2)
C8—C9—Fe1	70.81 (13)	C65—C64—H64	118.9
C10—C9—Fe1	70.31 (13)	C63—C64—H64	118.9
C8—C9—H9	126.3	C66—C65—C64	119.18 (19)
C10—C9—H9	126.3	C66—C65—H65	120.4
Fe1—C9—H9	124.2	C64—C65—H65	120.4
C6—C10—C9	107.2 (2)	C65—C66—C67	120.0 (2)
C6—C10—Fe1	71.05 (12)	C65—C66—H66	120.0
C9—C10—Fe1	68.95 (13)	C67—C66—H66	120.0
C6—C10—H10	126.4	C66—C67—C68	121.65 (19)
C9—C10—H10	126.4	C66—C67—H67	119.2
Fe1—C10—H10	125.2	C68—C67—H67	119.2
C12—C11—C16	118.05 (16)	C67—C68—C63	118.34 (17)
C12—C11—C1	117.74 (16)	C67—C68—P3	118.79 (14)
C16—C11—C1	124.01 (15)	C63—C68—P3	122.25 (14)
C13—C12—C11	122.65 (18)	C54—C69—C70	111.45 (14)
C13—C12—H12	118.7	C54—C69—P4	110.85 (13)
C11—C12—H12	118.7	C70—C69—P4	111.20 (12)
C12—C13—C14	119.43 (17)	C54—C69—H69	107.7
C12—C13—H13	120.3	C70—C69—H69	107.7
C14—C13—H13	120.3	P4—C69—H69	107.7
C15—C14—C13	119.06 (18)	C69—C70—H70A	109.5
C15—C14—H14	120.5	C69—C70—H70B	109.5
C13—C14—H14	120.5	H70A—C70—H70B	109.5
C14—C15—C16	122.55 (17)	C69—C70—H70C	109.5
C14—C15—H15	118.7	H70A—C70—H70C	109.5
C16—C15—H15	118.7	H70B—C70—H70C	109.5
C15—C16—C11	118.18 (16)	C76—C71—C72	118.06 (18)
C15—C16—P1	118.86 (13)	C76—C71—P3	123.41 (17)
C11—C16—P1	122.68 (13)	C72—C71—P3	118.52 (15)
C2—C17—C18	111.94 (14)	C73—C72—C71	121.1 (2)
C2—C17—P2	109.76 (12)	C73—C72—H72	119.4
C18—C17—P2	111.62 (12)	C71—C72—H72	119.4
C2—C17—H17	107.8	C74—C73—C72	120.2 (2)
C18—C17—H17	107.8	C74—C73—H73	119.9
P2—C17—H17	107.8	C72—C73—H73	119.9
C17—C18—H18A	109.5	C75—C74—C73	118.9 (2)
C17—C18—H18B	109.5	C75—C74—H74	120.5
H18A—C18—H18B	109.5	C73—C74—H74	120.5
C17—C18—H18C	109.5	C74—C75—C76	121.6 (2)
H18A—C18—H18C	109.5	C74—C75—H75	119.2
H18B—C18—H18C	109.5	C76—C75—H75	119.2
C24—C19—C20	118.39 (18)	C71—C76—C75	120.1 (2)
C24—C19—P1	122.67 (16)	C71—C76—H76	119.9
C20—C19—P1	118.93 (15)	C75—C76—H76	119.9
C21—C20—C19	120.4 (2)	C82—C77—C78	118.92 (19)
C21—C20—H20	119.8	C82—C77—P3	119.80 (15)
C19—C20—H20	119.8	C78—C77—P3	121.21 (15)
C22—C21—C20	120.7 (2)	C79—C78—C77	120.1 (2)
C22—C21—H21	119.7	C79—C78—H78	120.0
C20—C21—H21	119.7	C77—C78—H78	120.0
C23—C22—C21	119.4 (2)	C78—C79—C80	120.60 (19)
C23—C22—H22	120.3	C78—C79—H79	119.7
C21—C22—H22	120.3	C80—C79—H79	119.7
C22—C23—C24	120.6 (2)	C81—C80—C79	119.7 (2)
C22—C23—H23	119.7	C81—C80—H80	120.2
C24—C23—H23	119.7	C79—C80—H80	120.2
C19—C24—C23	120.5 (2)	C80—C81—C82	119.4 (2)
C19—C24—H24	119.7	C80—C81—H81	120.3
C23—C24—H24	119.7	C82—C81—H81	120.3
C30—C25—C26	118.45 (17)	C77—C82—C81	121.26 (19)
C30—C25—P1	119.26 (14)	C77—C82—H82	119.4
C26—C25—P1	122.28 (15)	C81—C82—H82	119.4
C27—C26—C25	119.96 (19)	C84—C83—C88	118.19 (18)
C27—C26—H26	120.0	C84—C83—P4	120.90 (15)
C25—C26—H26	120.0	C88—C83—P4	120.69 (14)
C28—C27—C26	120.86 (19)	C85—C84—C83	120.5 (2)
C28—C27—H27	119.6	C85—C84—H84	119.8
C26—C27—H27	119.6	C83—C84—H84	119.8
C27—C28—C29	119.66 (19)	C84—C85—C86	120.7 (2)
C27—C28—H28	120.2	C84—C85—H85	119.6
C29—C28—H28	120.2	C86—C85—H85	119.6
C28—C29—C30	119.46 (19)	C87—C86—C85	119.18 (19)
C28—C29—H29	120.3	C87—C86—H86	120.4
C30—C29—H29	120.3	C85—C86—H86	120.4
C25—C30—C29	121.58 (18)	C88—C87—C86	120.5 (2)
C25—C30—H30	119.2	C88—C87—H87	119.8
C29—C30—H30	119.2	C86—C87—H87	119.8
C32—C31—C36	118.25 (18)	C87—C88—C83	120.89 (19)
C32—C31—P2	120.19 (15)	C87—C88—H88	119.6
C36—C31—P2	121.18 (14)	C83—C88—H88	119.6
C33—C32—C31	121.0 (2)	C90—C89—C94	118.53 (18)
C33—C32—H32	119.5	C90—C89—P4	119.89 (14)
C31—C32—H32	119.5	C94—C89—P4	121.05 (15)
C34—C33—C32	120.2 (2)	C91—C90—C89	120.89 (19)
C34—C33—H33	119.9	C91—C90—H90	119.6
C32—C33—H33	119.9	C89—C90—H90	119.6
C33—C34—C35	119.4 (2)	C92—C91—C90	119.5 (2)
C33—C34—H34	120.3	C92—C91—H91	120.2
C35—C34—H34	120.3	C90—C91—H91	120.2
C36—C35—C34	120.7 (2)	C91—C92—C93	120.6 (2)
C36—C35—H35	119.7	C91—C92—H92	119.7
C34—C35—H35	119.7	C93—C92—H92	119.7
C35—C36—C31	120.43 (19)	C92—C93—C94	119.7 (2)
C35—C36—H36	119.8	C92—C93—H93	120.1
C31—C36—H36	119.8	C94—C93—H93	120.1
C38—C37—C42	118.25 (17)	C93—C94—C89	120.58 (19)
C38—C37—P2	119.53 (14)	C93—C94—H94	119.7
C42—C37—P2	122.05 (14)	C89—C94—H94	119.7
C37—C38—C39	121.35 (18)	C100—C95—C96	117.83 (19)
C37—C38—H38	119.3	C100—C95—C101	122.97 (18)
C39—C38—H38	119.3	C96—C95—C101	119.19 (18)
C40—C39—C38	119.89 (19)	C100—C95—Ru2	70.35 (11)
C40—C39—H39	120.1	C96—C95—Ru2	69.63 (11)
C38—C39—H39	120.1	C101—C95—Ru2	131.57 (14)
C39—C40—C41	119.45 (18)	C97—C96—C95	120.9 (2)
C39—C40—H40	120.3	C97—C96—Ru2	71.77 (12)
C41—C40—H40	120.3	C95—C96—Ru2	74.12 (11)
C40—C41—C42	120.62 (19)	C97—C96—H96	119.5
C40—C41—H41	119.7	C95—C96—H96	119.5
C42—C41—H41	119.7	Ru2—C96—H96	126.5
C41—C42—C37	120.44 (19)	C96—C97—C98	121.9 (2)
C41—C42—H42	119.8	C96—C97—Ru2	72.14 (12)
C37—C42—H42	119.8	C98—C97—Ru2	74.44 (11)
C44—C43—C48	118.17 (18)	C96—C97—H97	119.1
C44—C43—C49	119.46 (19)	C98—C97—H97	119.1
C48—C43—C49	122.4 (2)	Ru2—C97—H97	126.3
C44—C43—Ru1	70.52 (10)	C97—C98—C99	117.01 (19)
C48—C43—Ru1	70.16 (11)	C97—C98—C104	121.0 (2)
C49—C43—Ru1	129.60 (14)	C99—C98—C104	121.6 (2)
C43—C44—C45	121.33 (19)	C97—C98—Ru2	69.67 (11)
C43—C44—Ru1	73.56 (11)	C99—C98—Ru2	68.75 (11)
C45—C44—Ru1	71.45 (11)	C104—C98—Ru2	139.28 (15)
C43—C44—H44	119.3	C98—C99—C100	121.41 (19)
C45—C44—H44	119.3	C98—C99—Ru2	75.15 (12)
Ru1—C44—H44	127.8	C100—C99—Ru2	72.95 (11)
C46—C45—C44	121.1 (2)	C98—C99—H99	119.3
C46—C45—Ru1	75.05 (12)	C100—C99—H99	119.3
C44—C45—Ru1	72.15 (11)	Ru2—C99—H99	124.2
C46—C45—H45	119.5	C95—C100—C99	120.59 (19)
C44—C45—H45	119.5	C95—C100—Ru2	74.11 (11)
Ru1—C45—H45	125.0	C99—C100—Ru2	70.17 (11)
C45—C46—C47	117.38 (19)	C95—C100—H100	119.7
C45—C46—C52	121.5 (2)	C99—C100—H100	119.7
C47—C46—C52	120.5 (2)	Ru2—C100—H100	128.2
C45—C46—Ru1	69.15 (11)	C95—C101—C102	113.59 (18)
C47—C46—Ru1	69.04 (11)	C95—C101—C103	107.79 (18)
C52—C46—Ru1	140.75 (15)	C102—C101—C103	111.0 (2)
C46—C47—C48	121.86 (19)	C95—C101—H101	108.1
C46—C47—Ru1	75.14 (12)	C102—C101—H101	108.1
C48—C47—Ru1	71.90 (11)	C103—C101—H101	108.1
C46—C47—H47	119.1	C101—C102—H102	109.5
C48—C47—H47	119.1	C101—C102—H103	109.5
Ru1—C47—H47	125.7	H102—C102—H103	109.5
C43—C48—C47	119.81 (19)	C101—C102—H104	109.5
C43—C48—Ru1	73.56 (11)	H102—C102—H104	109.5
C47—C48—Ru1	71.37 (11)	H103—C102—H104	109.5
C43—C48—H48	120.1	C101—C103—H105	109.5
C47—C48—H48	120.1	C101—C103—H106	109.5
Ru1—C48—H48	126.9	H105—C103—H106	109.5
C43—C49—C50	114.4 (2)	C101—C103—H107	109.5
C43—C49—C51	108.33 (19)	H105—C103—H107	109.5
C50—C49—C51	111.4 (2)	H106—C103—H107	109.5
C43—C49—H49	107.5	C98—C104—H108	109.5
C50—C49—H49	107.5	C98—C104—H109	109.5
C51—C49—H49	107.5	H108—C104—H109	109.5
C49—C50—H50A	109.5	C98—C104—H110	109.5
C49—C50—H50B	109.5	H108—C104—H110	109.5
H50A—C50—H50B	109.5	H109—C104—H110	109.5
C49—C50—H50C	109.5	F1—P5—F5	90.62 (10)
H50A—C50—H50C	109.5	F1—P5—F2	178.65 (10)
H50B—C50—H50C	109.5	F5—P5—F2	90.73 (11)
C49—C51—H51A	109.5	F1—P5—F6	89.38 (9)
C49—C51—H51B	109.5	F5—P5—F6	178.66 (9)
H51A—C51—H51B	109.5	F2—P5—F6	89.27 (9)
C49—C51—H51C	109.5	F1—P5—F4	89.45 (8)
H51A—C51—H51C	109.5	F5—P5—F4	90.68 (9)
H51B—C51—H51C	109.5	F2—P5—F4	90.45 (9)
C46—C52—H52A	109.5	F6—P5—F4	90.66 (9)
C46—C52—H52B	109.5	F1—P5—F3	90.63 (7)
H52A—C52—H52B	109.5	F5—P5—F3	89.29 (8)
C46—C52—H52C	109.5	F2—P5—F3	89.47 (8)
H52A—C52—H52C	109.5	F6—P5—F3	89.37 (8)
H52B—C52—H52C	109.5	F4—P5—F3	179.91 (11)
C99—Ru2—C97	64.83 (8)	F9—P6—F8	92.24 (12)
C99—Ru2—C96	76.85 (7)	F9—P6—F11	93.37 (12)
C97—Ru2—C96	36.08 (9)	F8—P6—F11	92.94 (11)
C99—Ru2—C100	36.88 (7)	F9—P6—F7	89.83 (11)
C97—Ru2—C100	76.43 (7)	F8—P6—F7	175.86 (13)
C96—Ru2—C100	64.61 (7)	F11—P6—F7	90.51 (10)
C99—Ru2—C98	36.10 (8)	F9—P6—F10	177.68 (12)
C97—Ru2—C98	35.89 (8)	F8—P6—F10	88.63 (11)
C96—Ru2—C98	64.86 (8)	F11—P6—F10	88.73 (11)
C100—Ru2—C98	65.28 (7)	F7—P6—F10	89.17 (10)
C99—Ru2—C95	65.24 (7)	F9—P6—F12	88.39 (12)
C97—Ru2—C95	64.96 (8)	F8—P6—F12	88.24 (11)
C96—Ru2—C95	36.25 (7)	F11—P6—F12	177.84 (12)
C100—Ru2—C95	35.54 (7)	F7—P6—F12	88.24 (10)
C98—Ru2—C95	76.67 (7)	F10—P6—F12	89.49 (11)
			
C47—Ru1—P1—C25	25.50 (12)	C95—Ru2—P3—C77	124.68 (10)
C45—Ru1—P1—C25	66.91 (9)	P4—Ru2—P3—C77	−78.30 (7)
C48—Ru1—P1—C25	108.16 (19)	Cl2—Ru2—P3—C77	−159.16 (7)
C44—Ru1—P1—C25	102.56 (8)	C99—Ru2—P3—C71	−92.12 (10)
C43—Ru1—P1—C25	125.00 (9)	C97—Ru2—P3—C71	−42.73 (9)
C46—Ru1—P1—C25	36.86 (9)	C96—Ru2—P3—C71	−8.83 (9)
P2—Ru1—P1—C25	−76.58 (7)	C100—Ru2—P3—C71	−32.7 (2)
Cl1—Ru1—P1—C25	−157.43 (7)	C98—Ru2—P3—C71	−75.54 (9)
C47—Ru1—P1—C19	−89.65 (11)	C95—Ru2—P3—C71	9.42 (10)
C45—Ru1—P1—C19	−48.24 (9)	P4—Ru2—P3—C71	166.44 (7)
C48—Ru1—P1—C19	−7.0 (2)	Cl2—Ru2—P3—C71	85.59 (7)
C44—Ru1—P1—C19	−12.59 (8)	C99—Ru2—P3—C68	152.91 (10)
C43—Ru1—P1—C19	9.85 (9)	C97—Ru2—P3—C68	−157.70 (10)
C46—Ru1—P1—C19	−78.29 (8)	C96—Ru2—P3—C68	−123.80 (10)
P2—Ru1—P1—C19	168.28 (7)	C100—Ru2—P3—C68	−147.6 (2)
Cl1—Ru1—P1—C19	87.42 (7)	C98—Ru2—P3—C68	169.48 (9)
C47—Ru1—P1—C16	154.90 (11)	C95—Ru2—P3—C68	−105.55 (10)
C45—Ru1—P1—C16	−163.69 (9)	P4—Ru2—P3—C68	51.47 (8)
C48—Ru1—P1—C16	−122.44 (19)	Cl2—Ru2—P3—C68	−29.39 (8)
C44—Ru1—P1—C16	−128.03 (9)	C99—Ru2—P4—C89	−34.36 (9)
C43—Ru1—P1—C16	−105.60 (9)	C97—Ru2—P4—C89	−2.65 (13)
C46—Ru1—P1—C16	166.26 (9)	C96—Ru2—P4—C89	−87.72 (16)
P2—Ru1—P1—C16	52.83 (7)	C100—Ru2—P4—C89	−71.06 (9)
Cl1—Ru1—P1—C16	−28.03 (7)	C98—Ru2—P4—C89	−6.71 (9)
C47—Ru1—P2—C37	−44.47 (9)	C95—Ru2—P4—C89	−94.84 (9)
C45—Ru1—P2—C37	−4.42 (12)	P3—Ru2—P4—C89	104.07 (7)
C48—Ru1—P2—C37	−80.25 (9)	Cl2—Ru2—P4—C89	−176.90 (7)
C44—Ru1—P2—C37	−76.30 (17)	C99—Ru2—P4—C83	81.08 (8)
C43—Ru1—P2—C37	−100.23 (9)	C97—Ru2—P4—C83	112.78 (13)
C46—Ru1—P2—C37	−14.52 (9)	C96—Ru2—P4—C83	27.72 (17)
P1—Ru1—P2—C37	101.19 (7)	C100—Ru2—P4—C83	44.37 (8)
Cl1—Ru1—P2—C37	179.79 (7)	C98—Ru2—P4—C83	108.72 (9)
C47—Ru1—P2—C31	68.61 (9)	C95—Ru2—P4—C83	20.59 (9)
C45—Ru1—P2—C31	108.67 (11)	P3—Ru2—P4—C83	−140.49 (7)
C48—Ru1—P2—C31	32.84 (8)	Cl2—Ru2—P4—C83	−61.47 (7)
C44—Ru1—P2—C31	36.79 (17)	C99—Ru2—P4—C69	−161.22 (9)
C43—Ru1—P2—C31	12.85 (9)	C97—Ru2—P4—C69	−129.52 (13)
C46—Ru1—P2—C31	98.56 (9)	C96—Ru2—P4—C69	145.42 (16)
P1—Ru1—P2—C31	−145.72 (6)	C100—Ru2—P4—C69	162.07 (9)
Cl1—Ru1—P2—C31	−67.13 (7)	C98—Ru2—P4—C69	−133.58 (9)
C47—Ru1—P2—C17	−172.58 (9)	C95—Ru2—P4—C69	138.29 (9)
C45—Ru1—P2—C17	−132.53 (11)	P3—Ru2—P4—C69	−22.80 (7)
C48—Ru1—P2—C17	151.64 (9)	Cl2—Ru2—P4—C69	56.23 (7)
C44—Ru1—P2—C17	155.59 (16)	C56—Fe2—C53—C57	38.20 (12)
C43—Ru1—P2—C17	131.66 (9)	C61—Fe2—C53—C57	−30.4 (2)
C46—Ru1—P2—C17	−142.63 (9)	C62—Fe2—C53—C57	−73.46 (15)
P1—Ru1—P2—C17	−26.92 (7)	C55—Fe2—C53—C57	82.43 (12)
Cl1—Ru1—P2—C17	51.68 (7)	C60—Fe2—C53—C57	146.9 (4)
C9—Fe1—C1—C5	−37.6 (2)	C58—Fe2—C53—C57	−118.78 (13)
C4—Fe1—C1—C5	38.05 (11)	C59—Fe2—C53—C57	−162.09 (14)
C10—Fe1—C1—C5	−79.62 (14)	C54—Fe2—C53—C57	120.01 (16)
C3—Fe1—C1—C5	82.22 (11)	C56—Fe2—C53—C54	−81.81 (12)
C6—Fe1—C1—C5	−124.69 (12)	C57—Fe2—C53—C54	−120.01 (16)
C7—Fe1—C1—C5	−166.20 (13)	C61—Fe2—C53—C54	−150.41 (19)
C2—Fe1—C1—C5	119.92 (15)	C62—Fe2—C53—C54	166.53 (12)
C9—Fe1—C1—C2	−157.52 (16)	C55—Fe2—C53—C54	−37.58 (11)
C5—Fe1—C1—C2	−119.92 (15)	C60—Fe2—C53—C54	26.9 (4)
C4—Fe1—C1—C2	−81.87 (11)	C58—Fe2—C53—C54	121.21 (13)
C10—Fe1—C1—C2	160.45 (12)	C59—Fe2—C53—C54	77.90 (15)
C3—Fe1—C1—C2	−37.70 (10)	C56—Fe2—C53—C63	154.31 (19)
C6—Fe1—C1—C2	115.38 (12)	C57—Fe2—C53—C63	116.1 (2)
C7—Fe1—C1—C2	73.88 (15)	C61—Fe2—C53—C63	85.7 (3)
C9—Fe1—C1—C11	77.9 (2)	C62—Fe2—C53—C63	42.7 (2)
C5—Fe1—C1—C11	115.5 (2)	C55—Fe2—C53—C63	−161.45 (19)
C4—Fe1—C1—C11	153.54 (18)	C60—Fe2—C53—C63	−97.0 (4)
C10—Fe1—C1—C11	35.9 (2)	C58—Fe2—C53—C63	−2.7 (2)
C3—Fe1—C1—C11	−162.29 (18)	C59—Fe2—C53—C63	−46.0 (2)
C6—Fe1—C1—C11	−9.20 (19)	C54—Fe2—C53—C63	−123.9 (2)
C7—Fe1—C1—C11	−50.7 (2)	C57—C53—C54—C55	0.7 (2)
C2—Fe1—C1—C11	−124.6 (2)	C63—C53—C54—C55	−177.06 (18)
C5—C1—C2—C3	0.41 (19)	Fe2—C53—C54—C55	57.99 (13)
C11—C1—C2—C3	−178.62 (17)	C57—C53—C54—C69	−179.72 (17)
Fe1—C1—C2—C3	57.73 (11)	C63—C53—C54—C69	2.5 (3)
C5—C1—C2—C17	178.26 (15)	Fe2—C53—C54—C69	−122.46 (18)
C11—C1—C2—C17	−0.8 (3)	C57—C53—C54—Fe2	−57.26 (13)
Fe1—C1—C2—C17	−124.42 (17)	C63—C53—C54—Fe2	124.95 (19)
C5—C1—C2—Fe1	−57.32 (12)	C56—Fe2—C54—C55	−37.88 (12)
C11—C1—C2—Fe1	123.65 (18)	C57—Fe2—C54—C55	−82.06 (12)
C9—Fe1—C2—C3	12.8 (4)	C61—Fe2—C54—C55	22.2 (3)
C5—Fe1—C2—C3	−81.92 (12)	C62—Fe2—C54—C55	−154.8 (2)
C4—Fe1—C2—C3	−37.94 (11)	C60—Fe2—C54—C55	68.44 (15)
C10—Fe1—C2—C3	−162.64 (19)	C58—Fe2—C54—C55	157.59 (13)
C8—Fe1—C2—C3	64.63 (15)	C59—Fe2—C54—C55	113.11 (13)
C6—Fe1—C2—C3	152.39 (12)	C53—Fe2—C54—C55	−119.33 (16)
C7—Fe1—C2—C3	108.13 (13)	C56—Fe2—C54—C53	81.45 (12)
C1—Fe1—C2—C3	−119.25 (15)	C57—Fe2—C54—C53	37.27 (11)
C9—Fe1—C2—C1	132.0 (3)	C61—Fe2—C54—C53	141.5 (2)
C5—Fe1—C2—C1	37.33 (10)	C62—Fe2—C54—C53	−35.5 (3)
C4—Fe1—C2—C1	81.31 (11)	C55—Fe2—C54—C53	119.33 (16)
C10—Fe1—C2—C1	−43.4 (2)	C60—Fe2—C54—C53	−172.23 (13)
C3—Fe1—C2—C1	119.25 (15)	C58—Fe2—C54—C53	−83.08 (15)
C8—Fe1—C2—C1	−176.11 (13)	C59—Fe2—C54—C53	−127.56 (13)
C6—Fe1—C2—C1	−88.35 (13)	C56—Fe2—C54—C69	−157.51 (18)
C7—Fe1—C2—C1	−132.62 (12)	C57—Fe2—C54—C69	158.32 (18)
C9—Fe1—C2—C17	−106.6 (4)	C61—Fe2—C54—C69	−97.5 (3)
C5—Fe1—C2—C17	158.73 (17)	C62—Fe2—C54—C69	85.5 (3)
C4—Fe1—C2—C17	−157.29 (18)	C55—Fe2—C54—C69	−119.6 (2)
C10—Fe1—C2—C17	78.0 (3)	C60—Fe2—C54—C69	−51.2 (2)
C3—Fe1—C2—C17	−119.3 (2)	C58—Fe2—C54—C69	38.0 (2)
C8—Fe1—C2—C17	−54.7 (2)	C59—Fe2—C54—C69	−6.52 (19)
C6—Fe1—C2—C17	33.05 (19)	C53—Fe2—C54—C69	121.0 (2)
C7—Fe1—C2—C17	−11.22 (19)	C53—C54—C55—C56	0.1 (2)
C1—Fe1—C2—C17	121.4 (2)	C69—C54—C55—C56	−179.48 (17)
C1—C2—C3—C4	0.73 (19)	Fe2—C54—C55—C56	59.11 (14)
C17—C2—C3—C4	−177.14 (16)	C53—C54—C55—Fe2	−59.04 (13)
Fe1—C2—C3—C4	59.55 (12)	C69—C54—C55—Fe2	121.41 (18)
C1—C2—C3—Fe1	−58.82 (11)	C57—Fe2—C55—C56	−38.12 (12)
C17—C2—C3—Fe1	123.32 (16)	C61—Fe2—C55—C56	69.62 (14)
C9—Fe1—C3—C4	65.08 (15)	C62—Fe2—C55—C56	39.9 (3)
C5—Fe1—C3—C4	−37.78 (11)	C60—Fe2—C55—C56	111.01 (13)
C10—Fe1—C3—C4	36.7 (3)	C58—Fe2—C55—C56	177.6 (2)
C8—Fe1—C3—C4	106.19 (13)	C59—Fe2—C55—C56	150.84 (13)
C6—Fe1—C3—C4	176.21 (18)	C53—Fe2—C55—C56	−81.89 (13)
C7—Fe1—C3—C4	146.79 (12)	C54—Fe2—C55—C56	−119.29 (16)
C1—Fe1—C3—C4	−81.58 (11)	C56—Fe2—C55—C54	119.29 (16)
C2—Fe1—C3—C4	−119.06 (15)	C57—Fe2—C55—C54	81.17 (12)
C9—Fe1—C3—C2	−175.87 (13)	C61—Fe2—C55—C54	−171.08 (11)
C5—Fe1—C3—C2	81.27 (11)	C62—Fe2—C55—C54	159.2 (2)
C4—Fe1—C3—C2	119.06 (15)	C60—Fe2—C55—C54	−129.70 (12)
C10—Fe1—C3—C2	155.8 (2)	C58—Fe2—C55—C54	−63.2 (3)
C8—Fe1—C3—C2	−134.75 (13)	C59—Fe2—C55—C54	−89.87 (13)
C6—Fe1—C3—C2	−64.7 (2)	C53—Fe2—C55—C54	37.40 (11)
C7—Fe1—C3—C2	−94.15 (13)	C54—C55—C56—C57	−0.9 (2)
C1—Fe1—C3—C2	37.48 (10)	Fe2—C55—C56—C57	59.86 (14)
C2—C3—C4—C5	−1.6 (2)	C54—C55—C56—Fe2	−60.72 (13)
Fe1—C3—C4—C5	59.42 (13)	C61—Fe2—C56—C57	117.04 (13)
C2—C3—C4—Fe1	−61.03 (12)	C62—Fe2—C56—C57	78.62 (14)
C9—Fe1—C4—C5	110.90 (14)	C55—Fe2—C56—C57	−118.66 (17)
C10—Fe1—C4—C5	73.73 (14)	C60—Fe2—C56—C57	156.65 (12)
C3—Fe1—C4—C5	−119.09 (15)	C58—Fe2—C56—C57	63.6 (3)
C8—Fe1—C4—C5	151.60 (12)	C59—Fe2—C56—C57	176.70 (17)
C6—Fe1—C4—C5	66.6 (3)	C53—Fe2—C56—C57	−37.83 (11)
C7—Fe1—C4—C5	175.39 (15)	C54—Fe2—C56—C57	−81.23 (12)
C1—Fe1—C4—C5	−37.89 (10)	C57—Fe2—C56—C55	118.66 (17)
C2—Fe1—C4—C5	−81.41 (11)	C61—Fe2—C56—C55	−124.30 (13)
C9—Fe1—C4—C3	−130.01 (13)	C62—Fe2—C56—C55	−162.72 (12)
C5—Fe1—C4—C3	119.09 (15)	C60—Fe2—C56—C55	−84.69 (13)
C10—Fe1—C4—C3	−167.18 (12)	C58—Fe2—C56—C55	−177.7 (2)
C8—Fe1—C4—C3	−89.31 (13)	C59—Fe2—C56—C55	−64.6 (2)
C6—Fe1—C4—C3	−174.3 (3)	C53—Fe2—C56—C55	80.83 (12)
C7—Fe1—C4—C3	−65.52 (19)	C54—Fe2—C56—C55	37.43 (11)
C1—Fe1—C4—C3	81.20 (11)	C55—C56—C57—C53	1.3 (2)
C2—Fe1—C4—C3	37.68 (10)	Fe2—C56—C57—C53	61.49 (14)
C3—C4—C5—C1	1.9 (2)	C55—C56—C57—Fe2	−60.18 (14)
Fe1—C4—C5—C1	61.53 (12)	C54—C53—C57—C56	−1.3 (2)
C3—C4—C5—Fe1	−59.67 (13)	C63—C53—C57—C56	176.66 (17)
C2—C1—C5—C4	−1.4 (2)	Fe2—C53—C57—C56	−59.94 (14)
C11—C1—C5—C4	177.69 (16)	C54—C53—C57—Fe2	58.68 (13)
Fe1—C1—C5—C4	−60.22 (13)	C63—C53—C57—Fe2	−123.40 (17)
C2—C1—C5—Fe1	58.81 (12)	C61—Fe2—C57—C56	−75.86 (14)
C11—C1—C5—Fe1	−122.09 (16)	C62—Fe2—C57—C56	−117.77 (13)
C9—Fe1—C5—C4	−81.48 (14)	C55—Fe2—C57—C56	38.10 (12)
C10—Fe1—C5—C4	−124.16 (13)	C60—Fe2—C57—C56	−46.1 (2)
C3—Fe1—C5—C4	37.88 (11)	C58—Fe2—C57—C56	−156.03 (13)
C8—Fe1—C5—C4	−50.95 (19)	C59—Fe2—C57—C56	−173.9 (3)
C6—Fe1—C5—C4	−160.25 (12)	C53—Fe2—C57—C56	118.69 (16)
C1—Fe1—C5—C4	118.73 (15)	C54—Fe2—C57—C56	81.69 (12)
C2—Fe1—C5—C4	81.63 (11)	C56—Fe2—C57—C53	−118.69 (16)
C9—Fe1—C5—C1	159.79 (12)	C61—Fe2—C57—C53	165.45 (12)
C4—Fe1—C5—C1	−118.73 (15)	C62—Fe2—C57—C53	123.54 (13)
C10—Fe1—C5—C1	117.11 (13)	C55—Fe2—C57—C53	−80.59 (12)
C3—Fe1—C5—C1	−80.85 (11)	C60—Fe2—C57—C53	−164.79 (16)
C8—Fe1—C5—C1	−169.68 (14)	C58—Fe2—C57—C53	85.28 (14)
C6—Fe1—C5—C1	81.02 (14)	C59—Fe2—C57—C53	67.4 (4)
C2—Fe1—C5—C1	−37.10 (10)	C54—Fe2—C57—C53	−37.00 (11)
C9—Fe1—C6—C7	81.17 (16)	C56—Fe2—C58—C59	139.7 (2)
C5—Fe1—C6—C7	−178.48 (13)	C57—Fe2—C58—C59	−173.24 (13)
C4—Fe1—C6—C7	129.0 (3)	C61—Fe2—C58—C59	81.03 (15)
C10—Fe1—C6—C7	119.8 (2)	C62—Fe2—C58—C59	119.3 (2)
C3—Fe1—C6—C7	−42.1 (2)	C55—Fe2—C58—C59	−35.9 (3)
C8—Fe1—C6—C7	36.97 (15)	C60—Fe2—C58—C59	37.12 (14)
C1—Fe1—C6—C7	−133.39 (13)	C53—Fe2—C58—C59	−127.98 (13)
C2—Fe1—C6—C7	−87.91 (15)	C54—Fe2—C58—C59	−83.30 (16)
C9—Fe1—C6—C10	−38.66 (16)	C56—Fe2—C58—C62	20.4 (3)
C5—Fe1—C6—C10	61.69 (17)	C57—Fe2—C58—C62	67.48 (17)
C4—Fe1—C6—C10	9.2 (4)	C61—Fe2—C58—C62	−38.26 (14)
C3—Fe1—C6—C10	−161.90 (18)	C55—Fe2—C58—C62	−155.2 (2)
C8—Fe1—C6—C10	−82.86 (16)	C60—Fe2—C58—C62	−82.17 (16)
C7—Fe1—C6—C10	−119.8 (2)	C59—Fe2—C58—C62	−119.3 (2)
C1—Fe1—C6—C10	106.78 (14)	C53—Fe2—C58—C62	112.74 (14)
C2—Fe1—C6—C10	152.26 (13)	C54—Fe2—C58—C62	157.42 (13)
C10—C6—C7—C8	−0.4 (2)	C62—C58—C59—C60	0.4 (2)
Fe1—C6—C7—C8	−59.00 (15)	Fe2—C58—C59—C60	−58.46 (15)
C10—C6—C7—Fe1	58.60 (15)	C62—C58—C59—Fe2	58.83 (15)
C9—Fe1—C7—C8	37.85 (16)	C56—Fe2—C59—C60	−28.8 (3)
C4—Fe1—C7—C8	−35.8 (2)	C57—Fe2—C59—C60	142.4 (3)
C10—Fe1—C7—C8	82.23 (17)	C61—Fe2—C59—C60	38.05 (14)
C3—Fe1—C7—C8	−79.45 (16)	C62—Fe2—C59—C60	82.09 (15)
C6—Fe1—C7—C8	119.6 (2)	C55—Fe2—C59—C60	−73.83 (15)
C1—Fe1—C7—C8	−168.55 (13)	C58—Fe2—C59—C60	119.77 (19)
C2—Fe1—C7—C8	−125.20 (14)	C53—Fe2—C59—C60	−163.23 (13)
C9—Fe1—C7—C6	−81.74 (16)	C54—Fe2—C59—C60	−119.28 (13)
C4—Fe1—C7—C6	−155.41 (15)	C56—Fe2—C59—C58	−148.59 (19)
C10—Fe1—C7—C6	−37.36 (14)	C57—Fe2—C59—C58	22.6 (4)
C3—Fe1—C7—C6	160.96 (12)	C61—Fe2—C59—C58	−81.72 (15)
C8—Fe1—C7—C6	−119.6 (2)	C62—Fe2—C59—C58	−37.68 (14)
C1—Fe1—C7—C6	71.86 (17)	C55—Fe2—C59—C58	166.40 (13)
C2—Fe1—C7—C6	115.21 (13)	C60—Fe2—C59—C58	−119.77 (19)
C6—C7—C8—C9	0.6 (2)	C53—Fe2—C59—C58	77.00 (16)
Fe1—C7—C8—C9	−58.85 (16)	C54—Fe2—C59—C58	120.96 (14)
C6—C7—C8—Fe1	59.48 (15)	C58—C59—C60—C61	−0.7 (2)
C9—Fe1—C8—C7	−119.4 (2)	Fe2—C59—C60—C61	−59.63 (15)
C5—Fe1—C8—C7	−165.83 (14)	C58—C59—C60—Fe2	58.89 (15)
C4—Fe1—C8—C7	160.69 (13)	C56—Fe2—C60—C59	166.06 (13)
C10—Fe1—C8—C7	−80.62 (16)	C57—Fe2—C60—C59	−162.42 (17)
C3—Fe1—C8—C7	117.57 (14)	C61—Fe2—C60—C59	−118.7 (2)
C6—Fe1—C8—C7	−36.90 (14)	C62—Fe2—C60—C59	−80.87 (15)
C2—Fe1—C8—C7	79.69 (16)	C55—Fe2—C60—C59	123.46 (14)
C5—Fe1—C8—C9	−46.4 (2)	C58—Fe2—C60—C59	−37.08 (14)
C4—Fe1—C8—C9	−79.90 (15)	C53—Fe2—C60—C59	63.2 (4)
C10—Fe1—C8—C9	38.78 (15)	C54—Fe2—C60—C59	84.71 (16)
C3—Fe1—C8—C9	−123.03 (14)	C56—Fe2—C60—C61	−75.28 (16)
C6—Fe1—C8—C9	82.50 (16)	C57—Fe2—C60—C61	−43.8 (2)
C7—Fe1—C8—C9	119.4 (2)	C62—Fe2—C60—C61	37.79 (15)
C2—Fe1—C8—C9	−160.91 (13)	C55—Fe2—C60—C61	−117.88 (15)
C7—C8—C9—C10	−0.6 (2)	C58—Fe2—C60—C61	81.58 (16)
Fe1—C8—C9—C10	−61.13 (15)	C59—Fe2—C60—C61	118.7 (2)
C7—C8—C9—Fe1	60.52 (16)	C53—Fe2—C60—C61	−178.1 (3)
C5—Fe1—C9—C8	153.89 (13)	C54—Fe2—C60—C61	−156.63 (13)
C4—Fe1—C9—C8	112.74 (14)	C59—C60—C61—C62	0.8 (2)
C10—Fe1—C9—C8	−117.4 (2)	Fe2—C60—C61—C62	−59.96 (15)
C3—Fe1—C9—C8	75.54 (16)	C59—C60—C61—Fe2	60.80 (15)
C6—Fe1—C9—C8	−79.37 (15)	C56—Fe2—C61—C60	117.49 (15)
C7—Fe1—C9—C8	−36.73 (14)	C57—Fe2—C61—C60	157.67 (14)
C1—Fe1—C9—C8	179.24 (15)	C62—Fe2—C61—C60	−119.0 (2)
C2—Fe1—C9—C8	65.5 (4)	C55—Fe2—C61—C60	78.91 (16)
C5—Fe1—C9—C10	−88.67 (15)	C58—Fe2—C61—C60	−80.91 (16)
C4—Fe1—C9—C10	−129.82 (14)	C59—Fe2—C61—C60	−37.59 (15)
C3—Fe1—C9—C10	−167.03 (13)	C53—Fe2—C61—C60	179.00 (18)
C8—Fe1—C9—C10	117.4 (2)	C54—Fe2—C61—C60	62.5 (3)
C6—Fe1—C9—C10	38.06 (14)	C56—Fe2—C61—C62	−123.49 (14)
C7—Fe1—C9—C10	80.70 (15)	C57—Fe2—C61—C62	−83.30 (15)
C1—Fe1—C9—C10	−63.3 (2)	C55—Fe2—C61—C62	−162.07 (13)
C2—Fe1—C9—C10	−177.0 (3)	C60—Fe2—C61—C62	119.0 (2)
C7—C6—C10—C9	0.0 (2)	C58—Fe2—C61—C62	38.11 (14)
Fe1—C6—C10—C9	59.75 (15)	C59—Fe2—C61—C62	81.44 (15)
C7—C6—C10—Fe1	−59.73 (15)	C53—Fe2—C61—C62	−62.0 (2)
C8—C9—C10—C6	0.4 (2)	C54—Fe2—C61—C62	−178.4 (2)
Fe1—C9—C10—C6	−61.10 (15)	C60—C61—C62—C58	−0.6 (2)
C8—C9—C10—Fe1	61.45 (15)	Fe2—C61—C62—C58	−60.77 (15)
C9—Fe1—C10—C6	117.9 (2)	C60—C61—C62—Fe2	60.16 (15)
C5—Fe1—C10—C6	−136.35 (13)	C59—C58—C62—C61	0.1 (2)
C4—Fe1—C10—C6	−176.95 (12)	Fe2—C58—C62—C61	59.95 (15)
C3—Fe1—C10—C6	155.0 (2)	C59—C58—C62—Fe2	−59.80 (15)
C8—Fe1—C10—C6	79.28 (15)	C56—Fe2—C62—C61	70.16 (16)
C7—Fe1—C10—C6	36.56 (14)	C57—Fe2—C62—C61	111.78 (14)
C1—Fe1—C10—C6	−94.71 (14)	C55—Fe2—C62—C61	41.4 (3)
C2—Fe1—C10—C6	−64.0 (2)	C60—Fe2—C62—C61	−37.69 (14)
C5—Fe1—C10—C9	105.80 (15)	C58—Fe2—C62—C61	−118.2 (2)
C4—Fe1—C10—C9	65.20 (17)	C59—Fe2—C62—C61	−80.97 (15)
C3—Fe1—C10—C9	37.1 (3)	C53—Fe2—C62—C61	151.87 (13)
C8—Fe1—C10—C9	−38.57 (15)	C54—Fe2—C62—C61	178.4 (2)
C6—Fe1—C10—C9	−117.9 (2)	C56—Fe2—C62—C58	−171.64 (14)
C7—Fe1—C10—C9	−81.29 (16)	C57—Fe2—C62—C58	−130.02 (15)
C1—Fe1—C10—C9	147.43 (14)	C61—Fe2—C62—C58	118.2 (2)
C2—Fe1—C10—C9	178.11 (18)	C55—Fe2—C62—C58	159.58 (19)
C5—C1—C11—C12	71.9 (2)	C60—Fe2—C62—C58	80.51 (16)
C2—C1—C11—C12	−109.2 (2)	C59—Fe2—C62—C58	37.24 (15)
Fe1—C1—C11—C12	−14.6 (2)	C53—Fe2—C62—C58	−89.92 (16)
C5—C1—C11—C16	−103.0 (2)	C54—Fe2—C62—C58	−63.4 (3)
C2—C1—C11—C16	75.9 (2)	C57—C53—C63—C64	67.2 (3)
Fe1—C1—C11—C16	170.59 (13)	C54—C53—C63—C64	−115.3 (2)
C16—C11—C12—C13	−2.5 (3)	Fe2—C53—C63—C64	−20.4 (3)
C1—C11—C12—C13	−177.61 (18)	C57—C53—C63—C68	−107.9 (2)
C11—C12—C13—C14	0.1 (3)	C54—C53—C63—C68	69.5 (3)
C12—C13—C14—C15	2.2 (3)	Fe2—C53—C63—C68	164.47 (15)
C13—C14—C15—C16	−2.2 (3)	C68—C63—C64—C65	−2.5 (3)
C14—C15—C16—C11	−0.2 (3)	C53—C63—C64—C65	−177.9 (2)
C14—C15—C16—P1	173.89 (15)	C63—C64—C65—C66	−0.8 (4)
C12—C11—C16—C15	2.5 (3)	C64—C65—C66—C67	2.7 (4)
C1—C11—C16—C15	177.27 (17)	C65—C66—C67—C68	−1.3 (3)
C12—C11—C16—P1	−171.40 (14)	C66—C67—C68—C63	−2.1 (3)
C1—C11—C16—P1	3.4 (2)	C66—C67—C68—P3	169.10 (17)
C25—P1—C16—C15	−121.35 (15)	C64—C63—C68—C67	3.9 (3)
C19—P1—C16—C15	−15.31 (17)	C53—C63—C68—C67	179.02 (18)
Ru1—P1—C16—C15	102.82 (14)	C64—C63—C68—P3	−166.98 (15)
C25—P1—C16—C11	52.46 (16)	C53—C63—C68—P3	8.2 (3)
C19—P1—C16—C11	158.51 (15)	C77—P3—C68—C67	−121.93 (16)
Ru1—P1—C16—C11	−83.36 (16)	C71—P3—C68—C67	−15.37 (17)
C3—C2—C17—C18	−50.3 (2)	Ru2—P3—C68—C67	101.19 (15)
C1—C2—C17—C18	132.20 (17)	C77—P3—C68—C63	48.87 (17)
Fe1—C2—C17—C18	39.4 (2)	C71—P3—C68—C63	155.43 (16)
C3—C2—C17—P2	74.18 (19)	Ru2—P3—C68—C63	−88.01 (16)
C1—C2—C17—P2	−103.28 (17)	C55—C54—C69—C70	−52.6 (2)
Fe1—C2—C17—P2	163.89 (10)	C53—C54—C69—C70	127.88 (19)
C37—P2—C17—C2	−61.33 (13)	Fe2—C54—C69—C70	36.3 (2)
C31—P2—C17—C2	−168.11 (12)	C55—C54—C69—P4	71.8 (2)
Ru1—P2—C17—C2	73.60 (13)	C53—C54—C69—P4	−107.69 (18)
C37—P2—C17—C18	63.38 (14)	Fe2—C54—C69—P4	160.73 (10)
C31—P2—C17—C18	−43.40 (15)	C89—P4—C69—C54	−61.89 (13)
Ru1—P2—C17—C18	−161.69 (10)	C83—P4—C69—C54	−168.69 (12)
C25—P1—C19—C24	3.00 (19)	Ru2—P4—C69—C54	72.45 (13)
C16—P1—C19—C24	−102.10 (18)	C89—P4—C69—C70	62.68 (14)
Ru1—P1—C19—C24	127.89 (16)	C83—P4—C69—C70	−44.12 (15)
C25—P1—C19—C20	−177.58 (16)	Ru2—P4—C69—C70	−162.98 (10)
C16—P1—C19—C20	77.33 (17)	C77—P3—C71—C76	−1.18 (19)
Ru1—P1—C19—C20	−52.68 (17)	C68—P3—C71—C76	−104.92 (17)
C24—C19—C20—C21	−1.0 (3)	Ru2—P3—C71—C76	124.21 (16)
P1—C19—C20—C21	179.55 (17)	C77—P3—C71—C72	−179.89 (15)
C19—C20—C21—C22	−0.5 (3)	C68—P3—C71—C72	76.37 (16)
C20—C21—C22—C23	1.3 (4)	Ru2—P3—C71—C72	−54.50 (16)
C21—C22—C23—C24	−0.5 (4)	C76—C71—C72—C73	1.5 (3)
C20—C19—C24—C23	1.7 (3)	P3—C71—C72—C73	−179.70 (16)
P1—C19—C24—C23	−178.84 (17)	C71—C72—C73—C74	0.1 (3)
C22—C23—C24—C19	−1.0 (4)	C72—C73—C74—C75	−1.1 (3)
C19—P1—C25—C30	114.01 (16)	C73—C74—C75—C76	0.6 (4)
C16—P1—C25—C30	−141.76 (15)	C72—C71—C76—C75	−2.1 (3)
Ru1—P1—C25—C30	−2.91 (17)	P3—C71—C76—C75	179.19 (16)
C19—P1—C25—C26	−67.04 (18)	C74—C75—C76—C71	1.1 (3)
C16—P1—C25—C26	37.19 (18)	C71—P3—C77—C82	123.53 (17)
Ru1—P1—C25—C26	176.04 (14)	C68—P3—C77—C82	−132.22 (16)
C30—C25—C26—C27	1.0 (3)	Ru2—P3—C77—C82	8.00 (19)
P1—C25—C26—C27	−177.92 (16)	C71—P3—C77—C78	−59.44 (18)
C25—C26—C27—C28	0.8 (3)	C68—P3—C77—C78	44.81 (18)
C26—C27—C28—C29	−1.6 (3)	Ru2—P3—C77—C78	−174.97 (14)
C27—C28—C29—C30	0.6 (3)	C82—C77—C78—C79	0.2 (3)
C26—C25—C30—C29	−2.1 (3)	P3—C77—C78—C79	−176.88 (16)
P1—C25—C30—C29	176.90 (16)	C77—C78—C79—C80	0.8 (3)
C28—C29—C30—C25	1.3 (3)	C78—C79—C80—C81	−1.4 (3)
C37—P2—C31—C32	36.01 (17)	C79—C80—C81—C82	1.0 (4)
C17—P2—C31—C32	143.47 (15)	C78—C77—C82—C81	−0.6 (3)
Ru1—P2—C31—C32	−88.43 (15)	P3—C77—C82—C81	176.50 (17)
C37—P2—C31—C36	−151.15 (15)	C80—C81—C82—C77	0.0 (3)
C17—P2—C31—C36	−43.69 (17)	C89—P4—C83—C84	34.89 (17)
Ru1—P2—C31—C36	84.40 (16)	C69—P4—C83—C84	141.45 (15)
C36—C31—C32—C33	−0.6 (3)	Ru2—P4—C83—C84	−91.18 (15)
P2—C31—C32—C33	172.45 (17)	C89—P4—C83—C88	−150.60 (15)
C31—C32—C33—C34	0.2 (3)	C69—P4—C83—C88	−44.03 (17)
C32—C33—C34—C35	0.0 (4)	Ru2—P4—C83—C88	83.33 (15)
C33—C34—C35—C36	0.3 (4)	C88—C83—C84—C85	−0.8 (3)
C34—C35—C36—C31	−0.8 (3)	P4—C83—C84—C85	173.85 (15)
C32—C31—C36—C35	0.9 (3)	C83—C84—C85—C86	−0.8 (3)
P2—C31—C36—C35	−172.08 (16)	C84—C85—C86—C87	0.9 (3)
C31—P2—C37—C38	−149.01 (14)	C85—C86—C87—C88	0.7 (3)
C17—P2—C37—C38	101.35 (15)	C86—C87—C88—C83	−2.3 (3)
Ru1—P2—C37—C38	−34.41 (16)	C84—C83—C88—C87	2.3 (3)
C31—P2—C37—C42	35.86 (17)	P4—C83—C88—C87	−172.34 (15)
C17—P2—C37—C42	−73.79 (16)	C83—P4—C89—C90	−147.02 (16)
Ru1—P2—C37—C42	150.45 (13)	C69—P4—C89—C90	104.98 (16)
C42—C37—C38—C39	−1.2 (3)	Ru2—P4—C89—C90	−29.57 (18)
P2—C37—C38—C39	−176.48 (15)	C83—P4—C89—C94	41.39 (17)
C37—C38—C39—C40	0.6 (3)	C69—P4—C89—C94	−66.61 (17)
C38—C39—C40—C41	0.4 (3)	Ru2—P4—C89—C94	158.84 (13)
C39—C40—C41—C42	−0.8 (3)	C94—C89—C90—C91	2.3 (3)
C40—C41—C42—C37	0.3 (3)	P4—C89—C90—C91	−169.45 (17)
C38—C37—C42—C41	0.7 (3)	C89—C90—C91—C92	0.9 (3)
P2—C37—C42—C41	175.92 (15)	C90—C91—C92—C93	−2.9 (4)
C47—Ru1—C43—C44	101.15 (13)	C91—C92—C93—C94	1.6 (3)
C45—Ru1—C43—C44	28.99 (12)	C92—C93—C94—C89	1.7 (3)
C48—Ru1—C43—C44	131.30 (18)	C90—C89—C94—C93	−3.7 (3)
C46—Ru1—C43—C44	65.05 (12)	P4—C89—C94—C93	168.03 (15)
P2—Ru1—C43—C44	166.40 (9)	C99—Ru2—C95—C100	−29.57 (11)
P1—Ru1—C43—C44	−40.17 (13)	C97—Ru2—C95—C100	−102.03 (13)
Cl1—Ru1—C43—C44	−115.00 (11)	C96—Ru2—C95—C100	−131.41 (18)
C47—Ru1—C43—C48	−30.14 (12)	C98—Ru2—C95—C100	−65.95 (12)
C45—Ru1—C43—C48	−102.31 (13)	P4—Ru2—C95—C100	43.83 (13)
C44—Ru1—C43—C48	−131.30 (18)	P3—Ru2—C95—C100	−162.64 (9)
C46—Ru1—C43—C48	−66.25 (13)	Cl2—Ru2—C95—C100	123.69 (11)
P2—Ru1—C43—C48	35.11 (14)	C99—Ru2—C95—C96	101.84 (13)
P1—Ru1—C43—C48	−171.46 (10)	C97—Ru2—C95—C96	29.39 (13)
Cl1—Ru1—C43—C48	113.70 (12)	C100—Ru2—C95—C96	131.41 (18)
C47—Ru1—C43—C49	−146.2 (2)	C98—Ru2—C95—C96	65.46 (13)
C45—Ru1—C43—C49	141.6 (2)	P4—Ru2—C95—C96	175.25 (10)
C48—Ru1—C43—C49	−116.1 (3)	P3—Ru2—C95—C96	−31.23 (15)
C44—Ru1—C43—C49	112.6 (2)	Cl2—Ru2—C95—C96	−104.89 (12)
C46—Ru1—C43—C49	177.7 (2)	C99—Ru2—C95—C101	−146.7 (2)
P2—Ru1—C43—C49	−81.0 (2)	C97—Ru2—C95—C101	140.9 (2)
P1—Ru1—C43—C49	72.4 (2)	C96—Ru2—C95—C101	111.5 (2)
Cl1—Ru1—C43—C49	−2.4 (2)	C100—Ru2—C95—C101	−117.1 (2)
C48—C43—C44—C45	−2.1 (3)	C98—Ru2—C95—C101	176.9 (2)
C49—C43—C44—C45	179.38 (18)	P4—Ru2—C95—C101	−73.28 (19)
Ru1—C43—C44—C45	−55.40 (16)	P3—Ru2—C95—C101	80.3 (2)
C48—C43—C44—Ru1	53.29 (15)	Cl2—Ru2—C95—C101	6.59 (18)
C49—C43—C44—Ru1	−125.22 (17)	C100—C95—C96—C97	−3.7 (3)
C47—Ru1—C44—C43	−66.53 (12)	C101—C95—C96—C97	176.21 (18)
C45—Ru1—C44—C43	−132.13 (18)	Ru2—C95—C96—C97	−56.68 (17)
C48—Ru1—C44—C43	−29.40 (11)	C100—C95—C96—Ru2	53.00 (15)
C46—Ru1—C44—C43	−102.57 (13)	C101—C95—C96—Ru2	−127.11 (17)
P2—Ru1—C44—C43	−33.7 (2)	C99—Ru2—C96—C97	65.12 (13)
P1—Ru1—C44—C43	148.73 (11)	C100—Ru2—C96—C97	102.14 (14)
Cl1—Ru1—C44—C43	67.10 (11)	C98—Ru2—C96—C97	28.91 (12)
C47—Ru1—C44—C45	65.60 (14)	C95—Ru2—C96—C97	130.99 (19)
C48—Ru1—C44—C45	102.73 (14)	P4—Ru2—C96—C97	120.60 (15)
C43—Ru1—C44—C45	132.13 (18)	P3—Ru2—C96—C97	−71.21 (12)
C46—Ru1—C44—C45	29.55 (13)	Cl2—Ru2—C96—C97	−152.95 (12)
P2—Ru1—C44—C45	98.39 (19)	C99—Ru2—C96—C95	−65.88 (12)
P1—Ru1—C44—C45	−79.14 (12)	C97—Ru2—C96—C95	−130.99 (19)
Cl1—Ru1—C44—C45	−160.77 (12)	C100—Ru2—C96—C95	−28.85 (11)
C43—C44—C45—C46	−2.9 (3)	C98—Ru2—C96—C95	−102.08 (13)
Ru1—C44—C45—C46	−59.29 (16)	P4—Ru2—C96—C95	−10.4 (2)
C43—C44—C45—Ru1	56.38 (17)	P3—Ru2—C96—C95	157.79 (11)
C47—Ru1—C45—C46	28.61 (12)	Cl2—Ru2—C96—C95	76.06 (12)
C48—Ru1—C45—C46	65.36 (13)	C95—C96—C97—C98	−0.1 (3)
C44—Ru1—C45—C46	130.35 (19)	Ru2—C96—C97—C98	−57.85 (17)
C43—Ru1—C45—C46	101.73 (14)	C95—C96—C97—Ru2	57.80 (17)
P2—Ru1—C45—C46	−16.67 (17)	C99—Ru2—C97—C96	−102.58 (14)
P1—Ru1—C45—C46	−126.47 (12)	C100—Ru2—C97—C96	−65.31 (13)
Cl1—Ru1—C45—C46	157.44 (10)	C98—Ru2—C97—C96	−131.72 (19)
C47—Ru1—C45—C44	−101.74 (14)	C95—Ru2—C97—C96	−29.51 (12)
C48—Ru1—C45—C44	−64.99 (13)	P4—Ru2—C97—C96	−138.07 (11)
C43—Ru1—C45—C44	−28.62 (12)	P3—Ru2—C97—C96	112.02 (12)
C46—Ru1—C45—C44	−130.35 (19)	Cl2—Ru2—C97—C96	34.73 (15)
P2—Ru1—C45—C44	−147.02 (10)	C99—Ru2—C97—C98	29.14 (12)
P1—Ru1—C45—C44	103.18 (12)	C96—Ru2—C97—C98	131.72 (19)
Cl1—Ru1—C45—C44	27.09 (16)	C100—Ru2—C97—C98	66.41 (12)
C44—C45—C46—C47	6.8 (3)	C95—Ru2—C97—C98	102.21 (13)
Ru1—C45—C46—C47	−51.07 (16)	P4—Ru2—C97—C98	−6.35 (19)
C44—C45—C46—C52	−164.51 (19)	P3—Ru2—C97—C98	−116.26 (12)
Ru1—C45—C46—C52	137.60 (19)	Cl2—Ru2—C97—C98	166.45 (10)
C44—C45—C46—Ru1	57.89 (16)	C96—C97—C98—C99	5.3 (3)
C47—Ru1—C46—C45	−132.29 (18)	Ru2—C97—C98—C99	−51.49 (16)
C48—Ru1—C46—C45	−102.69 (14)	C96—C97—C98—C104	−167.1 (2)
C44—Ru1—C46—C45	−30.03 (12)	Ru2—C97—C98—C104	136.08 (19)
C43—Ru1—C46—C45	−66.08 (13)	C96—C97—C98—Ru2	56.78 (17)
P2—Ru1—C46—C45	169.32 (11)	C99—Ru2—C98—C97	−131.58 (19)
P1—Ru1—C46—C45	58.88 (13)	C96—Ru2—C98—C97	−29.05 (13)
Cl1—Ru1—C46—C45	−66.2 (3)	C100—Ru2—C98—C97	−101.27 (14)
C45—Ru1—C46—C47	132.29 (18)	C95—Ru2—C98—C97	−65.51 (13)
C48—Ru1—C46—C47	29.60 (12)	P4—Ru2—C98—C97	176.48 (11)
C44—Ru1—C46—C47	102.26 (13)	P3—Ru2—C98—C97	67.55 (12)
C43—Ru1—C46—C47	66.21 (13)	Cl2—Ru2—C98—C97	−34.7 (2)
P2—Ru1—C46—C47	−58.39 (12)	C97—Ru2—C98—C99	131.58 (19)
P1—Ru1—C46—C47	−168.83 (10)	C96—Ru2—C98—C99	102.53 (13)
Cl1—Ru1—C46—C47	66.0 (2)	C100—Ru2—C98—C99	30.31 (11)
C47—Ru1—C46—C52	113.0 (3)	C95—Ru2—C98—C99	66.07 (12)
C45—Ru1—C46—C52	−114.7 (3)	P4—Ru2—C98—C99	−51.94 (13)
C48—Ru1—C46—C52	142.6 (3)	P3—Ru2—C98—C99	−160.87 (11)
C44—Ru1—C46—C52	−144.7 (3)	Cl2—Ru2—C98—C99	96.9 (2)
C43—Ru1—C46—C52	179.2 (3)	C99—Ru2—C98—C104	114.2 (3)
P2—Ru1—C46—C52	54.6 (3)	C97—Ru2—C98—C104	−114.2 (3)
P1—Ru1—C46—C52	−55.8 (3)	C96—Ru2—C98—C104	−143.3 (3)
Cl1—Ru1—C46—C52	179.1 (2)	C100—Ru2—C98—C104	144.5 (3)
C45—C46—C47—C48	−6.0 (3)	C95—Ru2—C98—C104	−179.8 (3)
C52—C46—C47—C48	165.46 (19)	P4—Ru2—C98—C104	62.2 (3)
Ru1—C46—C47—C48	−57.09 (17)	P3—Ru2—C98—C104	−46.7 (3)
C45—C46—C47—Ru1	51.13 (16)	Cl2—Ru2—C98—C104	−148.9 (2)
C52—C46—C47—Ru1	−137.46 (18)	C97—C98—C99—C100	−6.9 (3)
C45—Ru1—C47—C46	−28.59 (11)	C104—C98—C99—C100	165.47 (19)
C48—Ru1—C47—C46	−131.40 (18)	Ru2—C98—C99—C100	−58.85 (16)
C44—Ru1—C47—C46	−65.30 (12)	C97—C98—C99—Ru2	51.93 (16)
C43—Ru1—C47—C46	−101.61 (13)	C104—C98—C99—Ru2	−135.69 (19)
P2—Ru1—C47—C46	125.32 (11)	C97—Ru2—C99—C98	−28.98 (12)
P1—Ru1—C47—C46	18.43 (17)	C96—Ru2—C99—C98	−65.16 (13)
Cl1—Ru1—C47—C46	−157.50 (9)	C100—Ru2—C99—C98	−130.19 (18)
C45—Ru1—C47—C48	102.80 (13)	C95—Ru2—C99—C98	−101.63 (13)
C44—Ru1—C47—C48	66.09 (12)	P4—Ru2—C99—C98	133.83 (12)
C43—Ru1—C47—C48	29.79 (12)	P3—Ru2—C99—C98	28.01 (15)
C46—Ru1—C47—C48	131.40 (18)	Cl2—Ru2—C99—C98	−148.32 (10)
P2—Ru1—C47—C48	−103.28 (12)	C97—Ru2—C99—C100	101.21 (13)
P1—Ru1—C47—C48	149.82 (10)	C96—Ru2—C99—C100	65.03 (12)
Cl1—Ru1—C47—C48	−26.11 (15)	C98—Ru2—C99—C100	130.19 (18)
C44—C43—C48—C47	3.0 (3)	C95—Ru2—C99—C100	28.56 (11)
C49—C43—C48—C47	−178.57 (18)	P4—Ru2—C99—C100	−95.98 (11)
Ru1—C43—C48—C47	56.43 (16)	P3—Ru2—C99—C100	158.20 (9)
C44—C43—C48—Ru1	−53.46 (16)	Cl2—Ru2—C99—C100	−18.13 (16)
C49—C43—C48—Ru1	125.00 (18)	C96—C95—C100—C99	2.0 (3)
C46—C47—C48—C43	1.1 (3)	C101—C95—C100—C99	−177.85 (18)
Ru1—C47—C48—C43	−57.49 (16)	Ru2—C95—C100—C99	54.69 (16)
C46—C47—C48—Ru1	58.61 (17)	C96—C95—C100—Ru2	−52.66 (15)
C47—Ru1—C48—C43	130.28 (18)	C101—C95—C100—Ru2	127.46 (18)
C45—Ru1—C48—C43	65.54 (12)	C98—C99—C100—C95	3.4 (3)
C44—Ru1—C48—C43	29.12 (11)	Ru2—C99—C100—C95	−56.54 (16)
C46—Ru1—C48—C43	101.37 (13)	C98—C99—C100—Ru2	59.91 (17)
P2—Ru1—C48—C43	−152.36 (11)	C99—Ru2—C100—C95	131.69 (18)
P1—Ru1—C48—C43	23.0 (3)	C97—Ru2—C100—C95	65.72 (12)
Cl1—Ru1—C48—C43	−68.27 (12)	C96—Ru2—C100—C95	29.40 (12)
C45—Ru1—C48—C47	−64.73 (13)	C98—Ru2—C100—C95	101.98 (13)
C44—Ru1—C48—C47	−101.16 (14)	P4—Ru2—C100—C95	−143.40 (11)
C43—Ru1—C48—C47	−130.28 (18)	P3—Ru2—C100—C95	55.4 (3)
C46—Ru1—C48—C47	−28.91 (12)	Cl2—Ru2—C100—C95	−59.84 (12)
P2—Ru1—C48—C47	77.36 (12)	C97—Ru2—C100—C99	−65.97 (13)
P1—Ru1—C48—C47	−107.28 (19)	C96—Ru2—C100—C99	−102.29 (13)
Cl1—Ru1—C48—C47	161.45 (11)	C98—Ru2—C100—C99	−29.71 (12)
C44—C43—C49—C50	168.95 (19)	C95—Ru2—C100—C99	−131.69 (18)
C48—C43—C49—C50	−9.5 (3)	P4—Ru2—C100—C99	84.91 (11)
Ru1—C43—C49—C50	80.7 (3)	P3—Ru2—C100—C99	−76.3 (2)
C44—C43—C49—C51	−66.2 (3)	Cl2—Ru2—C100—C99	168.47 (11)
C48—C43—C49—C51	115.3 (2)	C100—C95—C101—C102	−15.4 (3)
Ru1—C43—C49—C51	−154.51 (19)	C96—C95—C101—C102	164.71 (19)
C99—Ru2—P3—C77	23.14 (11)	Ru2—C95—C101—C102	76.9 (2)
C97—Ru2—P3—C77	72.53 (9)	C100—C95—C101—C103	108.0 (2)
C96—Ru2—P3—C77	106.43 (9)	C96—C95—C101—C103	−71.9 (2)
C100—Ru2—P3—C77	82.6 (2)	Ru2—C95—C101—C103	−159.75 (17)
C98—Ru2—P3—C77	39.71 (9)		

### Hydrogen-bond geometry (Å, °)

**Table:**

*D*—H···*A*	*D*—H	H···*A*	*D*···*A*	*D*—H···*A*
C20—H20···Cl1	0.95	2.69	3.499 (2)	143.
C72—H72···Cl2	0.95	2.56	3.389 (2)	145.
C13—H13···F3^i^	0.95	2.51	3.076 (2)	118.
C22—H22···F5^ii^	0.95	2.40	3.312 (3)	161.
C34—H34···F6^iii^	0.95	2.48	3.161 (3)	129.
C51—H51C···F9^iv^	0.98	2.54	3.422 (4)	149.
C60—H60···F6	0.95	2.55	3.380 (3)	146.
C65—H65···F11^v^	0.95	2.53	3.395 (3)	152.
C74—H74···F7^vi^	0.95	2.50	3.130 (3)	124.

Symmetry codes: (i) −*x*+1, *y*+1/2, −*z*; (ii) −*x*+2, *y*+1/2, −*z*; (iii) *x*, *y*+1, *z*; (iv) *x*+1, *y*, *z*; (v) −*x*+1, *y*−1/2, −*z*+1; (vi) −*x*, *y*−1/2, −*z*+1.
